# Shear behavior of single cast-in anchor simulating characteristics of bridge bearing anchor

**DOI:** 10.1038/s41598-022-17027-z

**Published:** 2022-08-03

**Authors:** Jin-Seok Choi, Won Jong Chin, Tian-Feng Yuan, Young-Soo Yoon

**Affiliations:** 1 School of Civil, Environmental, and Architectural Engineering, Korea University, 145 Anam-ro, Seongbuk-gu, Seoul, 02841 South Korea; 2 Department of Infrastructure Safety Research, Korea Institute of Civil Engineering and Building Technology, Goyang, 10223 South Korea

**Keywords:** Civil engineering, Structural materials

## Abstract

A bridge bearing anchor transmits various loads of a superstructure to a substructure. Most anchors are generally designed without consideration of characteristics such as concrete pedestal, grout bedding, and anchor socket. This study investigated the shear behavior of anchors in accordance with the edge distance, embedment depth, compressive strength of concrete, and height of the concrete pedestal in order to simulate the practical characteristics of the bridge bearing anchors. The actual shear capacity of the anchor differs from the shear strengths calculated by the ACI 318 and EN 1992-4; especially, the importance of the embedment depth is underestimated in these codes. An increase in the height of the concrete pedestal has a negative effect on the shear capacity because of the stress concentration. The grout is fractured prior to the occurrence of local damages in concrete, resulting in a secondary moment. As a result, the effect of the level arm is observed. An equation, which can predict the relative cracking degree of concrete, is proposed by analyzing the displacement of grout and concrete. High strain occurs in the stirrups close to the anchor, and the behavior of the strain is more influenced by the embedment depth than the edge distance. The comparison of obtained and analytically evaluated failure loads by calculations according to EN 1992-4, Schmid model and Sharma model was conducted to consider the effect of supplementary reinforcement. Finally, the design equation of concrete breakout strength is modified to predict the more precise shear resistance of a bridge bearing anchor.

## Introduction

General anchors under tensile and shear loads, which are eventually reflected in the design code, have been extensively studied^1–3^. Based on the databases developed by previous studies, anchors were introduced in ACI 349 (Appendix B)^4^. However, the ACI 349 presents an equation that directly correlates the failure behavior of an anchor system with its elastic and plastic behavior. This results in the overestimation of the fracture behavior of the anchor system rather than the actual behavior. Fuchs et al. proposed a concrete capacity design (CCD) method considering fracture properties of concrete^5^. Theoretical formula depending on the linear elastic fracture mechanics was reported based on the experimental results. The CCD method provides a theoretical background for the current ACI 318 and EN 1992-4, which contains concrete anchor-related design standards.

The behavior of high-strength anchors with large diameters has been extensively studied to further secure stability for their application in mainly nuclear power plants^6,7^. Besides various studies have been conducted on post-installed anchors that used for reconstruction and rehabilitation, as well as on cast-in-place anchors installed before the concrete is hardened^8,9^. The shear behavior of anchor groups having different configurations has also been thoroughly studied by considering various experimental studies^10–14^. Recently, many studies on anchors using fiber-reinforced polymer (FRP) reinforcing bars have been performed with the development of FRP technology^15^. In addition, various studies are also being performed such as the behavior of steel fiber-reinforced concrete (SFRC) anchor systems for improving the tensile strength of concrete and predicting the strength of anchors through machine running^16–18^. The shear and tensile behavior of single and anchor groups in SFRC are evaluated by giving appropriate design recommendations^19^.

**Figure 1:**
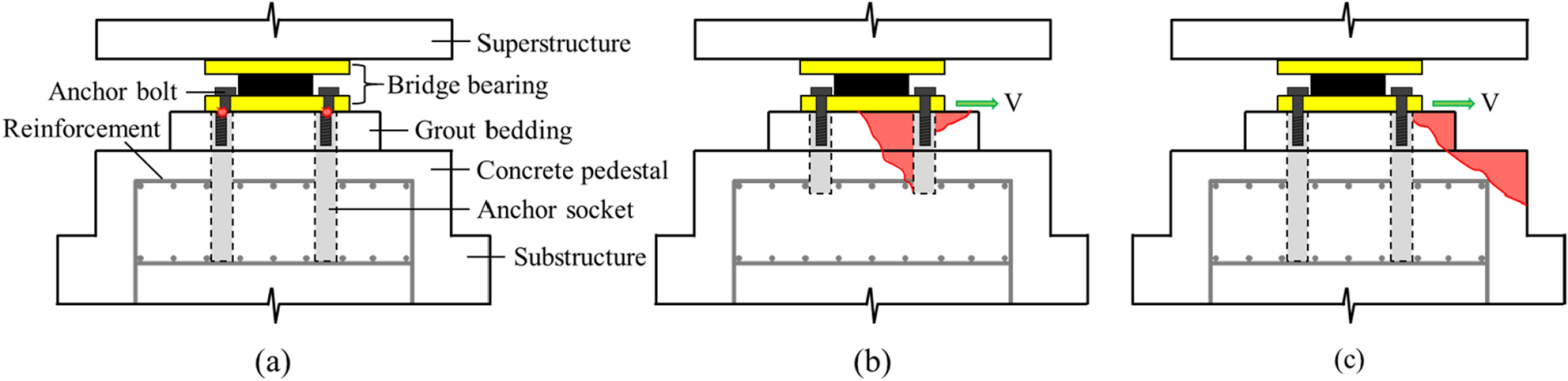
Schematic drawing of bridge bearing anchor and failure modes: (**a**) failure of anchor bolt, (**b**) concrete pryout, (**c**) concrete breakout.

A bridge bearing anchor is an important system to transfer various loads, such as vehicle and wind loads, from a superstructure to a substructure. The bearing must be completely connected so that the tensile stress, shear stress, and bending stress can be transferred to the concrete of the substructure through the bridge bearing anchor system^20^. The bridge bearing anchor exhibits characteristics such as a concrete pedestal installed on the substructure, grout bedding on the concrete pedestal, and anchor socket embedded in the concrete, as shown in Fig. 1^21^. Grout bedding levels any slope of the surface of abutment. It also protects the section of fixing bolts between the concrete and underside of the base plate^22^. The concrete pedestal is a small part compared to the abutment; however, it plays a major role as a structural member, which safely transmits the loads through the bearing capacity of the concrete. Although many studies about the concrete pedestal have been conducted, localized failures in bridge pedestal are yet to be clearly addressed^23^. In bridge bearing anchors, cast-in round steel sockets without spiral are mainly used. An anchor socket has the features of an easy-to-replace component after the anchor bolts have been damaged. Most of the previous studies have focused on anchors, which is directly embedded in concrete without anchor sockets; however, the shear behavior of anchors using anchor sockets has not yet been evaluated.

**Figure 2:**
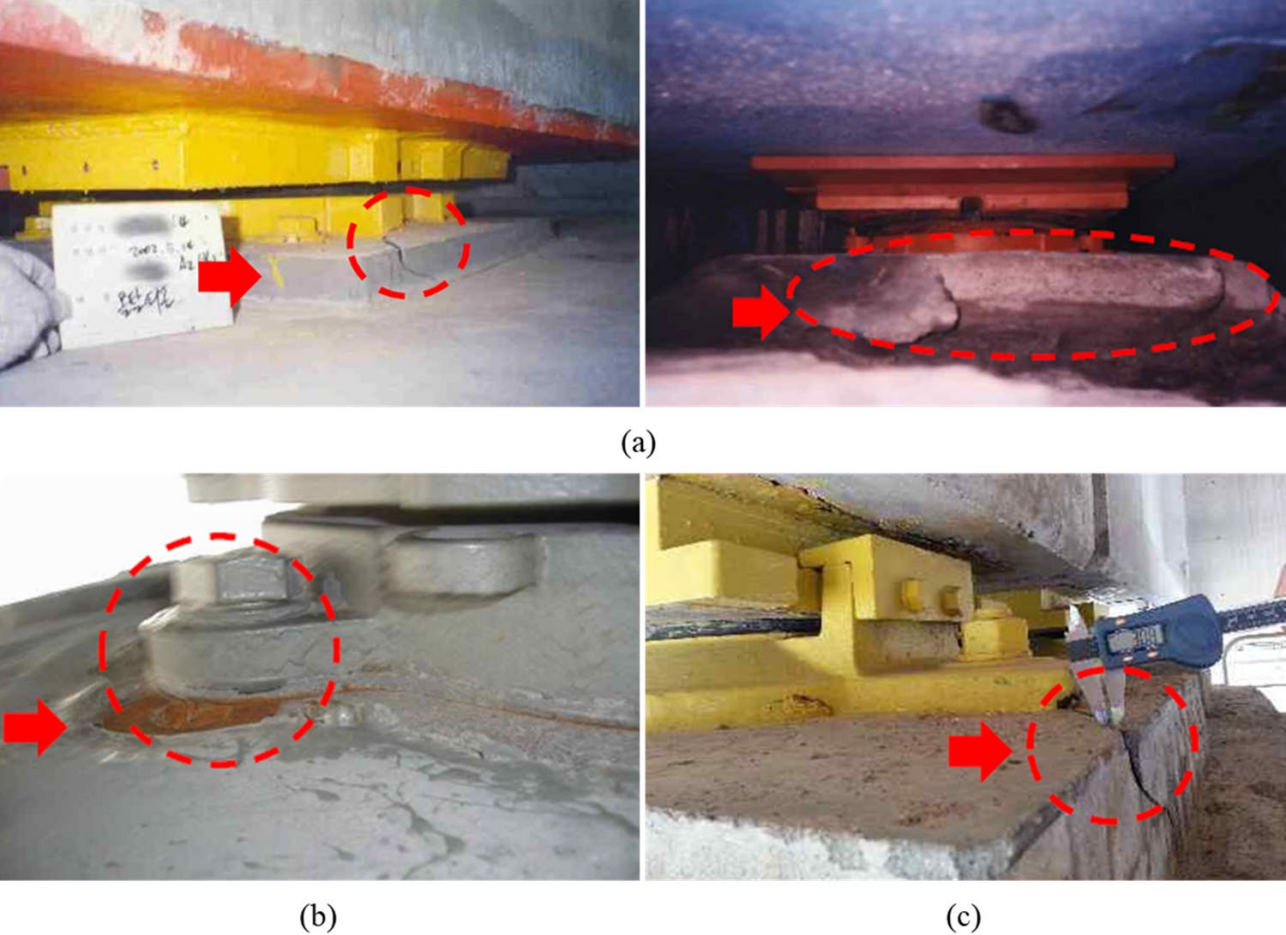
Damage types of bridge bearing anchors: (**a**) damage to the grout bedding, (**b**) failure of anchor bolt, (**c**) breakout failure of concrete pedestal.

Although bridge bearing anchors exhibit very different characteristics from general cast-in-place anchors, a bridge bearing anchor is designed based on the design code of a general anchor without considering any of the abovementioned characteristics. Thus many structural stability problems are encountered in bridge bearing anchors such as breakout failure of concrete pedestal, damage to grout bedding, and failure of anchor bolts, which are shown in Fig. 2^24^. Recently, many bridge bearing anchors were actually damaged by the Pohang earthquake in South Korea, which has lower peak ground acceleration (PGA) than design PGA of the bridges^25^. In many previous incidents, a bridge superstructure has collapsed due to earthquakes, such as the: San Fernado earthquake (1971), the Loma Prieta earthquake (1989), the Northridge Earthquake (1994), and the Kobe earthquake (1995)^26,27^. Because of these events, the philosophy of the seismic design of a bridge has changed from the only focus of strength to consideration of ductility, as well as importance of the bearing have begun to be highlighted^28,29^. Therefore, seismic isolation systems, such as isolation bearings and dampers, have been introduced resulting in improvement of resistance to seismic force; however, there is no reassessment of the design of bearing anchors.

Accordingly, this study aims to investigate the behavior of bridge bearing anchors under shear loading. The bridge bearing anchors considering the described features were tested under a quasi-static load. Following are the specific objectives of this study: (1) compare the experimental and predicted strength of the current code; (2) evaluate the effects of edge distance, embedment depth, compressive strength of concrete, and bearing height on shear capacity; (3) analyze the effects of grout bedding and stirrup reinforcement; and (4) modify the strength equation of the bridge bearing anchor.

## Experimental program

### Specimen details, manufacturing, and materials

**Figure 3:**
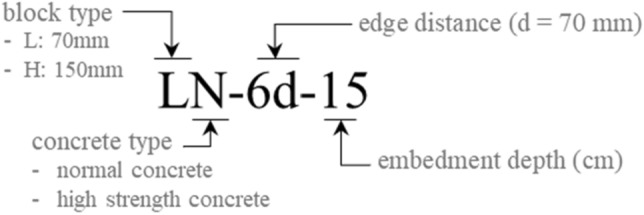
Designation of test specimens.

In this test program, a total of twenty-one specimens of bridge bearing anchors with seventeen variables were fabricated and tested under monotonic shear load. Four duplicated variables were set for validating the main variable in the concrete breakout capacity. The designation of the specimens reflects the edge distance, embedment depth of the anchor socket, height of the concrete pedestal, and compressive strength of concrete by the mixture. The details of each test variable are given below and listed in Table 1 and Fig. 3:

1. Height of concrete pedestal and grout bedding: The height of the concrete pedestal plays an important role in determining the load capacity of concrete pedestals. In this study, 70 and 150 mm heights were set as variables for the concrete pedestal, which are generally the pedestal heights depending on the space under the bridge superstructure^30^. For better stability, it is recommended that grout bedding height should not exceed 50 mm beyond the top of the concrete pedestal; thus, the height of the grout bedding was set to 50 mm^31^.
2. Edge distance: The minimum edge distance for a cast-in anchor without torsion is stipulated to satisfy the specified cover requirement for reinforcement in the ACI 318. The edge distance of the anchor is an important factor in supporting the bridge bearing system, which resists the horizontal load. To induce concrete breakout failure, the edge distance was set to 6d, 5d, 4.5d, 3.2d, and 2.7d, where d is the diameter of the anchor socket.
3. Embedment depth of anchor socket: It is recommended to ensure that the anchor socket reaches at least the upper surface of the reinforcement in the substructure. However, in many cases, the embedment depth is too short to avoid interference with the dense reinforcement. The embedment variables were established to determine its effect on the shear capacity and to confirm the bearing stress distribution along the anchor socket, which is indicated by the power term in the code. Here, embedment depth variables were set from the depth reaching the upper surface of the reinforcement in the substructure to the surface of the abutment.
4. Compressive strength of concrete pedestal and grout bedding: the substructure of abutment and the concrete pedestal are generally poured together with the same mixture. In bridge abutment, a compressive strength of 30 MPa is mostly used, followed by high-strength concrete of approximately 60 MPa strength. The mixture properties are listed in Table 2. In case of grout bedding, a ready-made product is used to protect against the vulnerability of connections and facilitate re-bed in the renovation of pedestals. In this study, the non-shrink grout was manufactured using a high-strength (target strength of 60 MPa) hydraulic-cementitious grout (produced by the Republic of Korea) at a *w/b* of 0.16.

**Table 1:** Test matrix.

Specimen ID	block type	${f}_{ck}$^a^ (MPa)	${f}_{g}$^b^ (MPa)	${c}_{a1}$^c^ (mm)	${l}_{e}$^d^ (mm)
LN-6d-15	L	36.2	66.6	420 (6d)	150
LN-6d-7	L	36.2	66.6	420 (6d)	70
LN-5d-15	L	24.9	61.6	350 (5d)	150
LN-5d-15 (2)	L	24.9	61.6	350 (5d)	150
LN-4.5d-22	L	36.2	66.6	315 (4.5d)	220
LN-4.5d-18	L	30.2	60.5	315 (4.5d)	180
LN-4.5d-15	L	30.2	60.5	315 (4.5d)	150
LN-4.5d-15 (2)	L	30.2	60.5	315 (4.5d)	150
LN-4.5d-11	L	30.2	60.5	315 (4.5d)	110
LN-4.5d-7	L	36.2	66.6	315 (4.5d)	110
LN-3.2d-15	L	30.2	60.5	224 (3.2d)	150
LN-3.2d-15 (2)	L	30.2	60.5	224 (3.2d)	150
LN-2.7d-15	L	24.9	61.6	189 (2.7d)	150
LH-4.5d-15	L	61.5	60.5	315 (4.5d)	150
LH-3.2d-15	L	61.5	60.5	224 (3.2d)	150
HN-6d-15	H	36.2	66.6	420 (6d)	150
HN-4.5d-30	H	36.2	66.6	315 (4.5d)	300
HN-4.5d-23	H	36.2	66.6	315 (4.5d)	230
HN-4.5d-15	H	36.2	66.6	315 (4.5d)	150
HN-4.5d-15 (2)	H	36.2	66.6	315 (4.5d)	150
HH-4.5d-15	H	61.5	60.5	315 (4.5d)	150

^a^Compressive strength of concrete at test day.

^b^Compressive strength of grout at test day.

^c^Distance from the center of an anchor shaft to the edge of concrete in one direction.

^d^Embedment depth of anchor socket.

**Table 2:** Mixture proportions of concrete (by unit weight).

	Unit weight (kg/m^3^)					${f}_{cr}$^a^ (MPa)
	Water	Cement	*w/b*	Fine aggregate	Coarse aggregate	
Normal concrete	160	279	0.57	811	948	30
High strength concrete	173	526	0.33	750	896	60

^a^Is the required compressive strength of concrete.

In a general bridge bearing anchor, reinforcement should be embedded in both concrete pedestal and substructure to obtain an additional confinement effect, which secures the bearing capacity of concrete and carries the shear capacity of the anchor steel. In this study, No. 5 bars with a nominal yield strength of Grade 60 steel were used with a horizontal spacing of 100 mm and vertical spacing of 120 mm. The reinforcement was bent 90° from top of the concrete pedestal and embedded in the substructure to satisfy the required development length. According to the tensile test of the bar, the yield strength and strain were 446.13 MPa and 2110 × 10^–6^ mm/mm, respectively.

**Figure 4:**
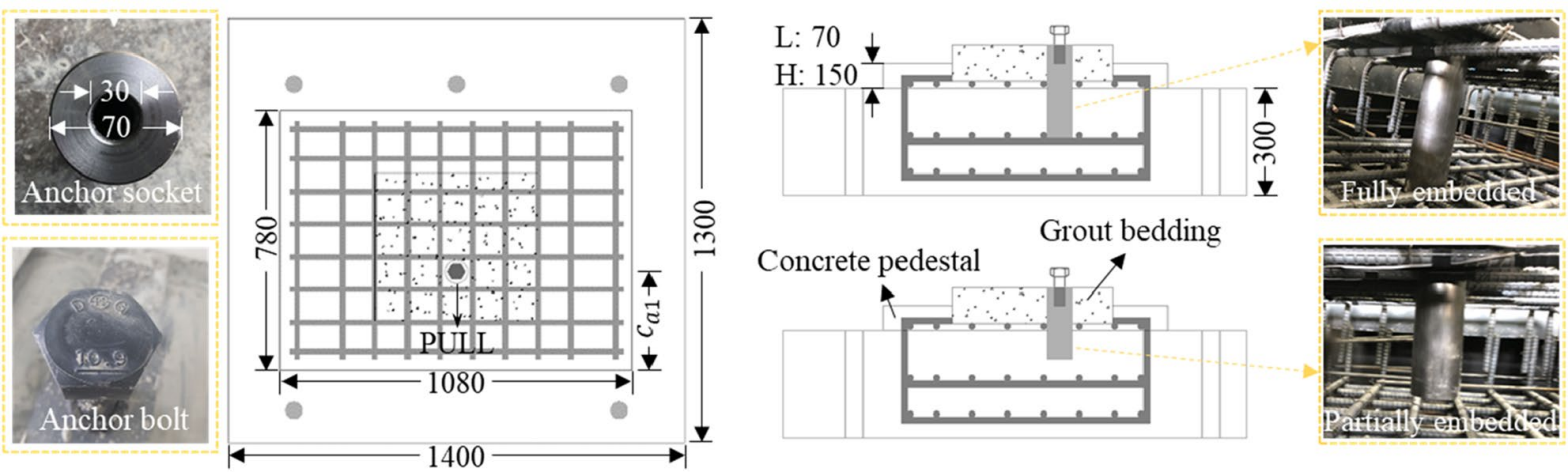
Geometric details of test specimens.

**Figure 5:**
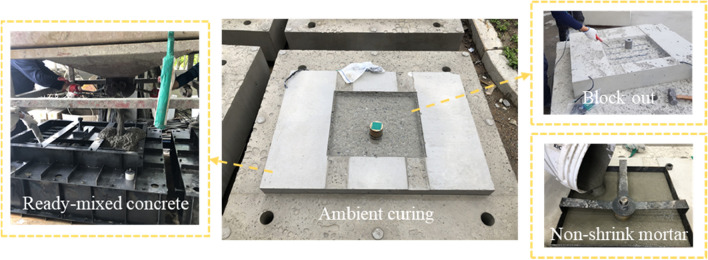
Manufacturing process of bridge bearing anchor.

In addition, a hex structural anchor bolt with a diameter of 30 mm was used, in accordance with ASTM A490M Grade 10.9 (${f}_{y}$ = 940 MPa, ${f}_{ut}$ = 1040 MPa), which is commonly used in bridge bearings^32^. For anchor sockets, the nominal yield strength of Grade 55 steel with a diameter (d) of 70 mm was used, which was designed to be engaged with anchor bolts with a screw line of 30 mm diameter and 50 mm length. All specimens had the same geometry, cross-sectional dimensions of 450 mm × 500 mm in the grout bedding section, 780 mm × 1080 mm in the concrete pedestal section, and 1400 mm × 1300 mm in the substructure section, as shown in Fig. 4. The manufacturing process of the specimens was performed in the steps, as shown in Fig. 5. After 3 days of ambient curing, the cover of the concrete pedestal was cut out by 50 mm up to reinforcement surface, and a high-strength non-shrink grout was placed in the cut space to fabricate the grout bedding.

### Test setup

**Figure 6:**
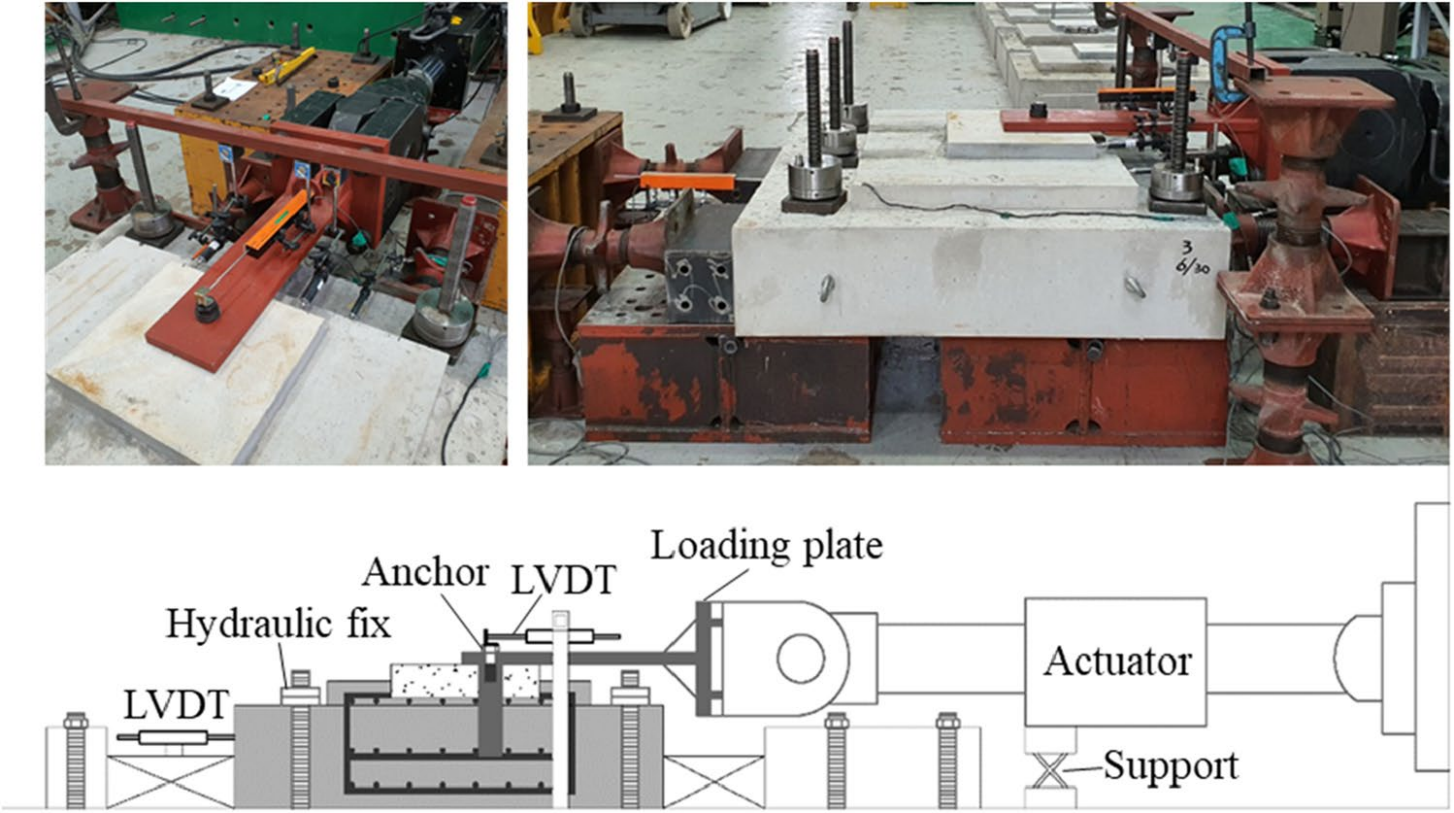
Test setup for monotonic shear load.

The monotonic shear performance of the bridge bearing anchor was evaluated through the setup shown in Fig. 6. During the specimen fabrication, five holes were created in advance to fix the specimen to the ground. First, the bottom of the specimen was fixed using a high-strength steel bar and nuts. Then, the specimen was slowly fixed with hydraulic fixes at the top with a capacity of 49 kN. Additional fixed supports were used on both sides of the loading direction to prevent a push toward the load direction. The loading plate, which was 30-mm-thick steel plate, was considered as the bottom plate of the actual bearing to tightly fasten the anchor bolt. A 980 kN actuator was used to apply monotonic shear loading to the anchor through the loading plate, at a displacement control rate of 1.2 mm/min. The actuator body was braced on a strong floor by using a support to prevent the rotation of the loading plate and the downward motion of the actuator head. A linear variable differential transformer (LVDT) was installed to measure the anchor displacement. As a result of analyzing displacement of the LVDT installed on the rear of the test specimen, no slip between the test specimen and floor was observed.

## Experimental results and discussion

### Typical behavior of anchors under monotonic shear loading

Table 3 lists the measured shear capacities and predicted strengths by the equations presented in both the ACI 318 and EN 1992-4 codes. The current design code does not directly reflect the grout bedding characteristics of the bridge bearing anchors, as it only deals with general concrete anchors. Therefore, in this study, the predicted strength was conservatively calculated for comparison with the measured strength by considering the strength of the grout placed inside the concrete pedestal to be equal the concrete strength. Table 3 shows that EN 1992-4 has more conservative approach to strength calculations when compare to ACI 318 across all test specimens, showing a difference in strength ranging from 11.7 to 35.2% with respect to concrete breakout strength. The three main types of failure modes observed for the anchors are represented in Table 3. The failure loads of the duplicated variables showed similar results except for the LN-5d-15 anchor.

**Table 3:** Comparison of test results and strength by ACI 318 and EN1992-4.

Specimen ID	${V}_{t}$^a^ (kN)	Calculated using ACI 318^b^^2^			Calculated using EN 1992-4^c^^3^			Failure mode^d^
		${V}_{cb}$ (kN)	${V}_{cp}$ (kN)	$\frac{{V}_{t}}{\mathrm{min}({V}_{cb}, {V}_{cp})}$	${V}_{Rk,c}$ (kN)	${V}_{Rk,cp}$ (kN)	$\frac{{V}_{t}}{\mathrm{min}({V}_{Rk,c}, {V}_{Rk,cp})}$	
LN-6d-15	394.4	326.6	340.4	1.21	272.3	259.4	1.52	SF
LN-6d-7	172.9	280.4	158.2	1.09	238.2	120.5	1.43	PO
LN-5d-15	258.8	276.9	282.3	0.93	243.8	215.1	1.20	CB
LN-5d-15 (2)	244.6	276.9	282.3	0.88	243.8	215.1	1.14	CB
LN-4.5d-22	340.1	263.0	468.1	1.29	223.2	356.7	1.52	CB
LN-4.5d-18	304.2	230.8	283.4	1.32	197.6	216.0	1.54	CB
LN-4.5d-15	246.8	222.5	310.9	1.11	189.6	236.9	1.30	CB
LN-4.5d-15 (2)	245.5	222.5	310.9	1.10	189.6	236.9	1.29	CB
LN-4.5d-11	200.9	209.1	222.4	0.96	177.7	169.5	1.19	CB
LN-4.5d-7	115.6	209.2	158.2	0.73	175.7	120.5	0.96	PO
LN-3.2d-15	179.4	150.8	271.1	1.19	139.3	206.6	1.29	CB
LN-3.2d-15 (2)	180.0	150.8	271.1	1.19	139.3	206.6	1.29	CB
LN-2.7d-15	113.9	106.1	219.0	1.07	104.7	166.9	1.09	CB
LH-4.5d-15	322.2	317.6	443.6	1.01	255.8	338.0	1.26	SF
LH-3.2d-15	260.6	215.2	386.9	1.21	187.9	294.8	1.39	CB
HN-6d-15	299.9	383.5	340.4	0.88	319.7	259.4	1.16	SF
HN-4.5d-30	334.9	308.6	554.9	1.09	294.8	422.8	1.14	SF
HN-4.5d-23	319.4	292.7	480.5	1.09	274.6	366.1	1.16	CB
HN-4.5d-15	226.2	265.3	310.9	0.85	224.9	236.9	1.01	CB
HN-4.5d-15 (2)	225.2	265.3	310.9	0.85	224.9	236.9	1.00	CB
HH-4.5d-15	293.3	350.2	443.6	0.84	224.9	338.0	1.30	CB

^a^Measured failure load.

^b^Calculated on the basis of ACI 318, ${V}_{cb}$ is the concrete breakout strength, ${V}_{cp}$ is the concrete pryout strength.

^c^Calculated on the basis of EN 1992-4, ${V}_{Rk,c}$ is the concrete breakout strength, ${V}_{Rk,c}$ is the concretepryout strength.

^*d*^*SF* Steel failure of anchor bolt, *PO* pryout failure, *CB* concrete breakout failure.

#### Effect of edge distance on shear behavior

**Figure 7:**
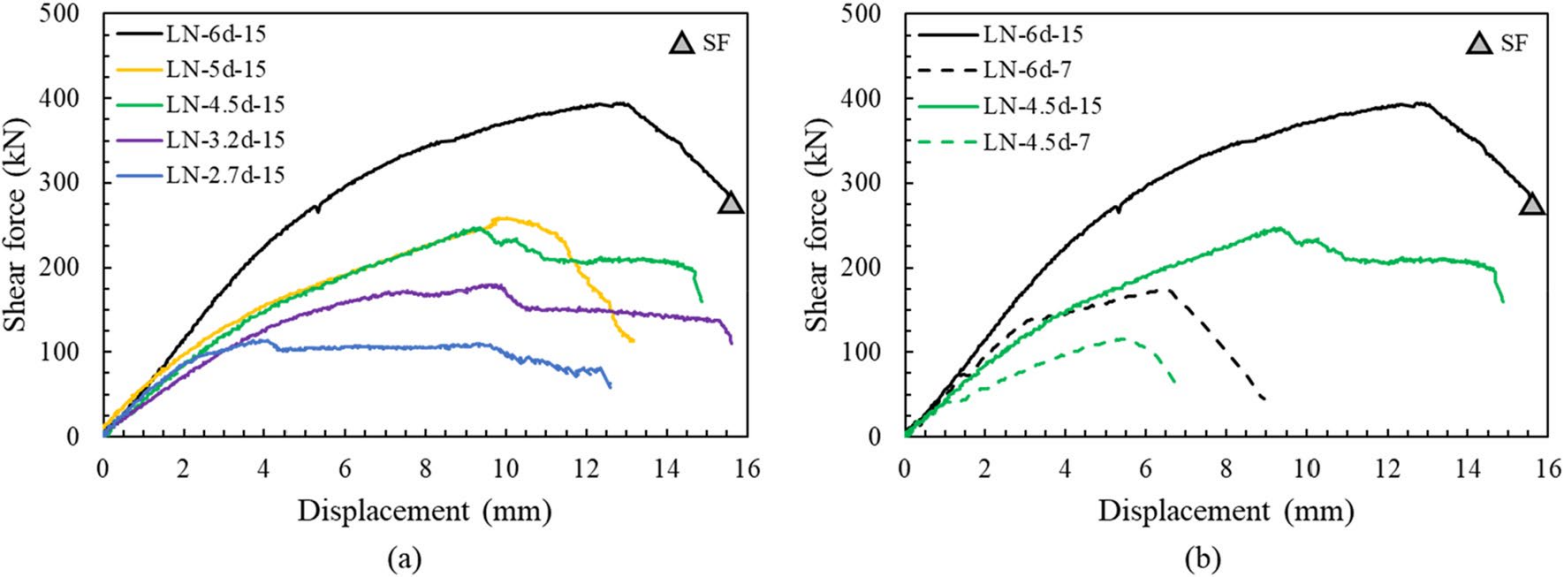
Load–displacement curve, edge distance: (**a**) embedment depth of 150 mm, (**b**) embedment depth of 70 mm.

**Figure 8:**
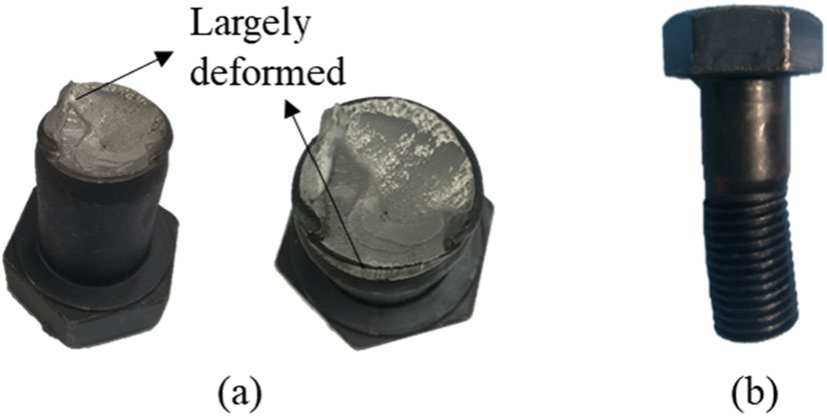
Failure shape of anchor bolt: (**a**) typical fracture, (**b**) bending.

Figure 7 shows the load displacement curve of the edge distance series, which display a positive relationship between an increase in shear capacity and edge distance. For the series of anchors with an embedment depth of 150 mm (Fig. 7a), only specimen LN-6d-15 failed by anchor shaft fracture (Fig. A.1), whereas the specimens with an edge distance of less than 6d failed by concrete breakout (Figs. A.3, A.7, A.11, and A.13). In the figure, SF indicates the steel failure of the anchor bolt. In this study, the anchor bolt was not directly embedded in the concrete, thus the load was transmitted to structure through the anchor socket. The anchor fracture started from a flexural crack of the bolt after reaching a maximum load and then the anchor did not exhibit ductile behavior after the peak load. The failure load exceeded far beyond the code-specified anchor shear capacity because it was mainly caused by the fracture of the anchor shaft largely, as shown in Fig. 8a^33^. The bearing anchors with edge distances of less than 5d showed slight ductility behavior after the peak load, unlike the LN-5d-15 anchor. In addition, only the measured shear capacity of the LN-5d-15 anchor was smaller than the predicted strengths by ACI 318. This is because the breakout strength and pryout strength calculated by the ACI 318 code are similar due to the sufficient edge distance of 5d. It is considered that this affects the difference in failure load only for LN-5D-15 specimen among duplicated specimens and lead to dissimilar crack pattern (Figs. A.3 and A.4). In addition, the grout bedding of the specimen with a relatively short edge distance than concrete initially resisted most of the load, thus cracks first occurred on the grout bedding. If the mortar is damaged, the capability to prevent the rotation and displacement of the anchor rod is lost^34^.

Anchors with an embedment depth of 70 mm exhibited different failure behaviors, as shown in Fig. 7b. The slope of the graph changed significantly after the initial cracking, and then rapidly decreased after the peak load. In addition, for the variable with an edge distance of 6d, the decrease in the embedment depth resulted in rear cracks rather than front cracks. The failure mode also changed from bolt failure to pryout failure (Figs. A.1, A.2). Similarly, rear cracks occurred more frequently compared to front cracks in the variable edge distance of 4.5d (Figs. A.7, A.8, A.10). This failure pattern was slightly different from the general pryout failure mode, because the socket was not pulled out by preventing the rotation of the anchor socket through the bearing effect of the reinforcing bar^35–37^. In accordance with the increase of the edge distance, the actual strength compared to the predicted pryout strength increased from 0.73 to 1.09 and from 0.96 to 1.43 when using ACI 318 and EN 1992-4, respectively. The shear capacity significantly decreased by 56.2% for 6d and by 53.0% for 4.5d by embedment depth decrease, since the governing failure mode was changed from breakout to pryout (Figs. A.1, A.2, A.7, A.8, A.10). These results indicate the importance of securing a sufficient embedment depth, as designated failure mode can be pre-planned by designing appropriate embedment depth to prevent sudden failure of anchor bolt or concrete pryout.

#### Effect of the embedment depth on shear behavior

**Figure 9:**
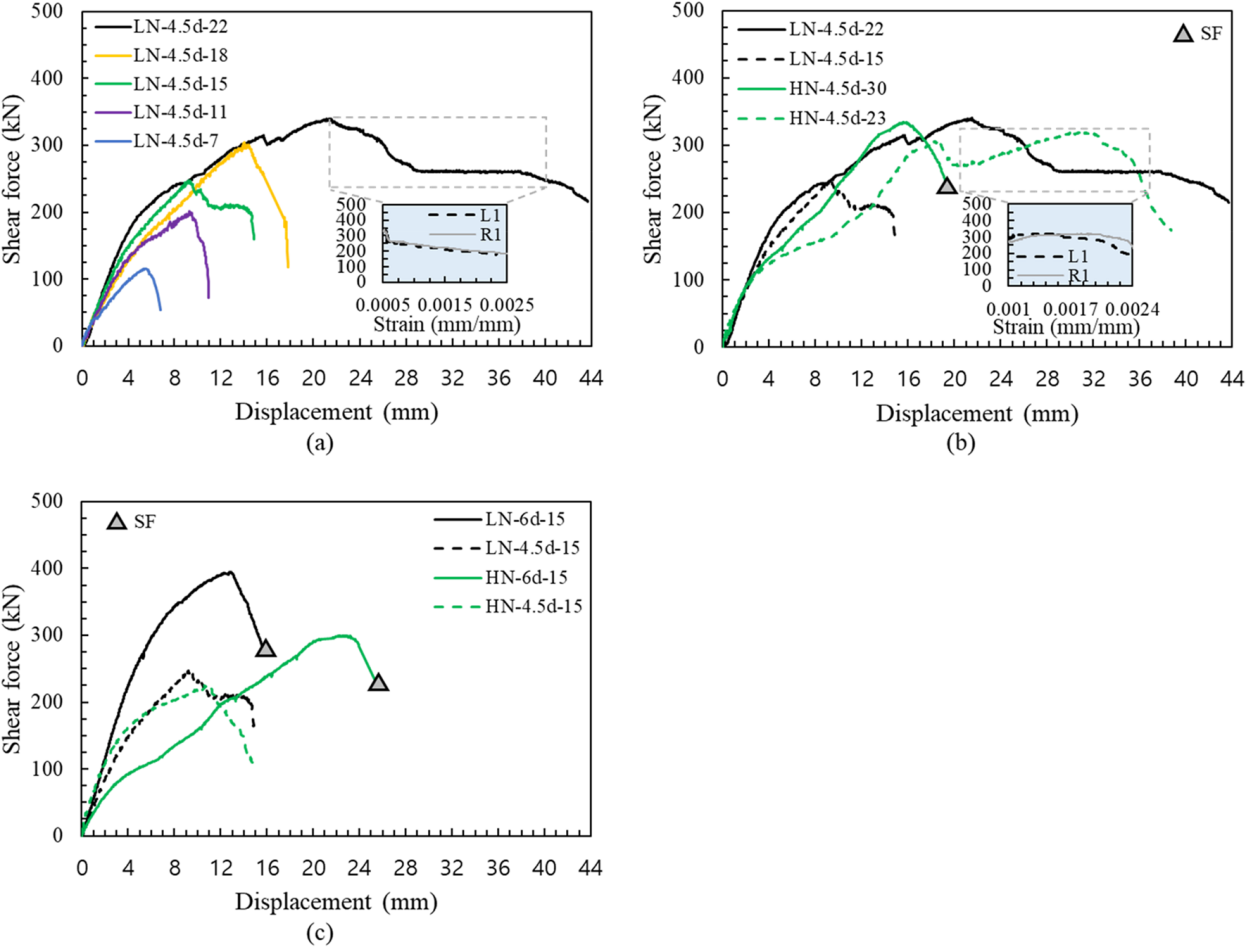
Load–displacement curve, embedment depth: (**a**) edge distance of 4.5d, (**b**) different embedment depth with two types of pedestal height, (**c**) same embedment depth with two types of pedestal height.

Figure 9a shows the load displacement curve according to embedment depth at an edge distance of 4.5d. Comparisons for height of the concrete pedestal with and without changing the embedment depth are shown in Fig. 9b,c, respectively. Figure 9b compares the variable in which the embedment depth increased by the same amount as increase of the height of the concrete pedestal. In Fig. 9c, the HN-6d-15 and HN-4.5d-15 specimens were not embedded into the substructure due to an increase in the height of the concrete pedestal, which has height of 150 mm.

According to most design codes of the anchor, such as ACI 318, EN 1992-4, ETAG 001, and KCI, the embedment depth is evaluated to have little influence on the breakout strength under shear with a power term set to just 0.2 or less^2,3,38,39^. However, these code equations were derived from the experimental results obtained for plain concrete without considering any reinforcements, which were embedded in the actual bridge bearing anchor. Klingner et al. reported an enhanced shear capacity of a reinforced anchor with a double layer of 180° hairpin^20^. Nilsson et al. reported that surface reinforcement significantly increased the bearing capacity of concrete, which is related to the embedment depth in the design code equation^40^. In the LN-4.5d series (Fig. 9a), both shear capacity and displacement at the peak load increased with increase of the embedment depth. The measured shear capacity was larger than the predicted strength for the specimens with embedment greater than 110 mm, where the variables have the ratio of embedment depth to anchor socket diameter exceeding 2. This is because the specimens with deeper embedment depth close to reinforcement can obtain the restraint effect from the reinforcement compared to specimens with shallow embedment. This result indicated that the bearing stress distribution was not transmitted well along the shaft of the anchor socket, as the ratio was less than 2^41^.

The LN-4.5d-22 specimen embedded up to the substructure reinforcement exhibited considerable ductility after the peak load. The shear resistance of the concrete and grout decreased after the peak load; however, the reinforcements next to the anchor (L1, R1) deformed beyond the yield strength and the ductility of the anchor system increased, which agree with the findings of Segle^42^. This result indicates that the shear capacity of the reinforced anchor is related to not only surface reinforcements in concrete pedestal but also reinforcements in substructure.

For all specimens with a concrete pedestal height of 150 mm and embedment depth of 150 mm, the shear capacities were measured to be smaller than the predicted strengths calculated using ACI 318 and close to the predicted strengths using EN 1992-4, as shown in Table 3. As indicated in Fig. 9b, the HN-4.5d-23 specimen in which the anchor socket was not sufficiently embedded to reinforcement in substructure showed an increase in shear capacity according to increase in embedment depth. The LN-4.5d-22 and HN-4.5d-23 specimens with similar embedment depths showed ductile behavior of the anchor bolts (Fig. 8b). The two specimens failed by concrete breakout (Figs. A.5, A.18), rather than bolt failure as the strain rate of reinforcement increased after peak load. Therefore, it should lead to destruction of concrete in advance to induce ductile behavior of bolts by setting the proper height of the pedestal and embedment depth.

For HN-4.5d-15 and HN-6d-15 specimens in Fig. 9c, the slope of the load displacement after the first crack rapidly decreased, and the maximum shear capacity also decreased in comparison with the low and high concrete pedestals. This is because the structural response of a concrete bearing depends not only on the surface area available to resist loading, but also on its height. Yahya reported that an increase in the pedestal height can improve the ductility of the pedestal under a low load; however, the overall stiffness is reduced^23^. This result is also attributed to the load not being well-transferred to the substructure but concentrated on the pedestal, as reported by the Korea Expressway Corporation^43^. Therefore, to minimize the stress concentration in the pedestal and to transfer the load well to the substructure, the edge distance needs to be increased with the increase of the height for proper bridge bearing anchor systems^30^.

#### *Effect of compressive strength of concrete on shear behavior*

**Figure 10:**
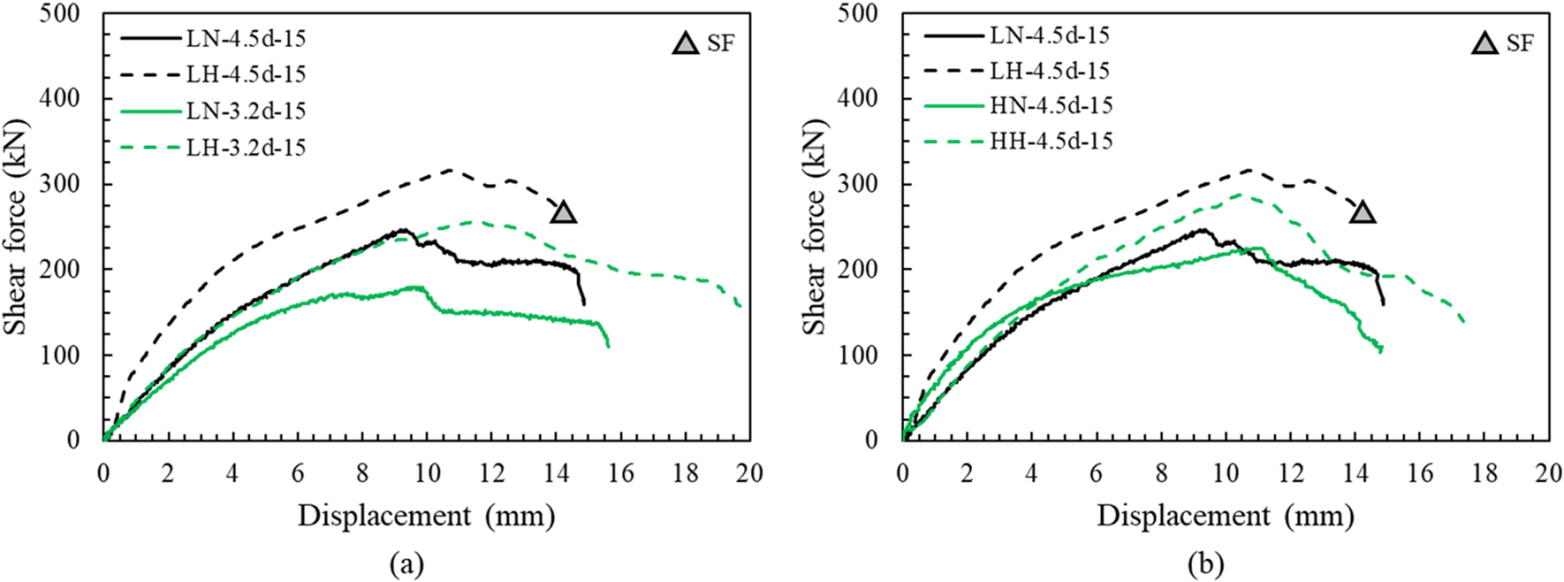
Load–displacement curve, compressive strength of concrete: (**a**) edge distance, (**b**) pedestal height.

According to the ACI 318 and EN 1992-4, the strength of an anchor is proportional to the square root of the compressive strength of concrete under both shear and tension. This is because the anchor uses the tensile strength of concrete, which is frequently assumed to be proportional to $\sqrt{{f}_{ck}}$ in concrete engineering^44^. The load displacement curves of the anchor for the concrete compressive strength were evaluated as shown in Fig. 10. As the compressive strength increased, the concrete edge breakout resistance of the bridge bearing anchor also increased. The ductile behavior after the peak was found to be similar regardless of strength because there was no improvement in the tensile strength as in the case of using fibers^45^. There were only few cracks in the concrete pedestal of LH-4.5d-15 specimen (Fig. A.14b), however the bolt was destroyed due to the high compressive strength of the concrete.

**Figure 11:**
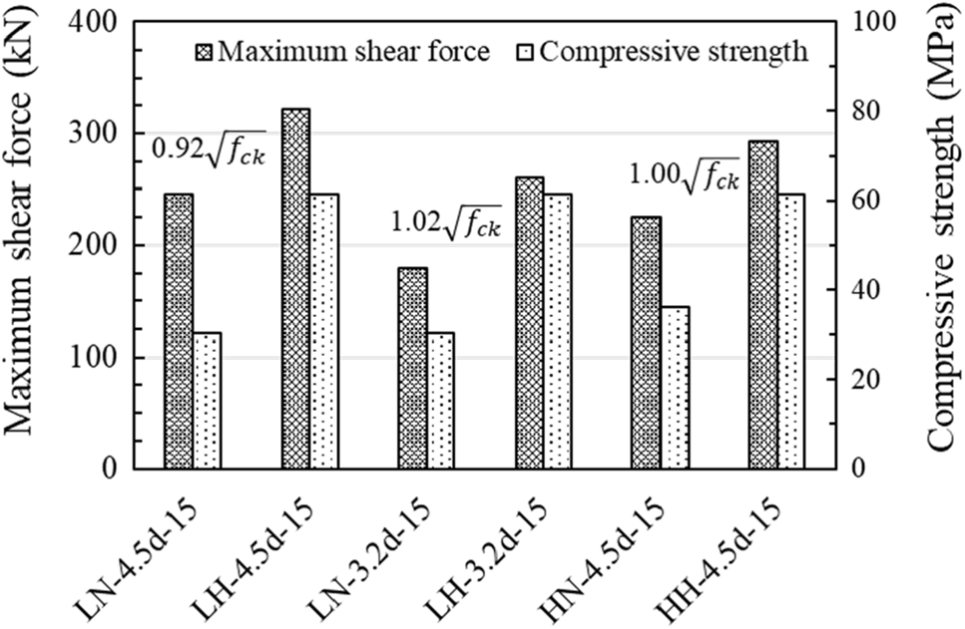
Comparison of maximum shear force due to two types of compressive strength of concrete.

As shown in Fig. 10b, the shear capacity decreased as the height of the concrete pedestal increased, irrespective of the compressive strength of the concrete. This is because anchor socket of the specimens with a low height of concrete pedestal was embedded into substructures that had relatively large volumes, whereas anchor socket of the specimens with higher concrete pedestals were embedded only in the pedestal. Figure 11 shows that the increase in the maximum shear force was almost proportional to that in the square root of the increase in compressive strength, regardless of the height of the concrete pedestal. This result is consistent with the suitability of the current code for anchors to use the tensile strength of concrete. Meanwhile, a comparison between LN-4.5d-15 and LH-4.5d-15 showed that the maximum shear force was proportional to 0.92 $\sqrt{{f}_{ck}}$ instead of 1.00 $\sqrt{{f}_{ck}}$ because LH-4.5d-15 failed by steel failure of the bolt rather than breakout failure. Therefore, it is concluded that regardless of the pedestal height, the square root in relation to concrete compressive strength in the current code does not need to be changed when calculating the maximum shear force.

### Effect of grout on shear behavior of bridge bearing anchor

#### Effect of level arm caused by critically damaged grout

**Figure 12:**
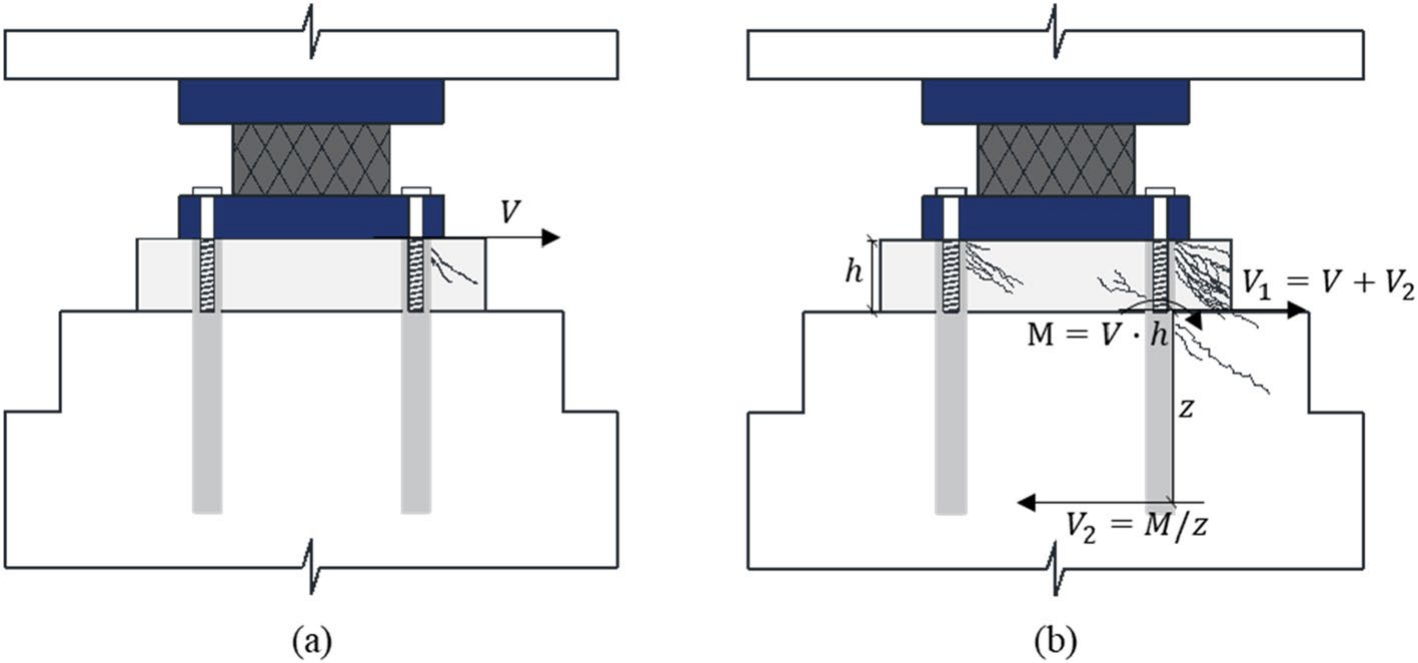
Damaged bridge bearing anchor: (**a**) w/o level arm, (**b**) w/ level arm.

The shear load beyond resistance capacity formed inclined failure surfaces both on the grout bedding and concrete pedestal. In some test specimens, the inclination of the main failure surface in the grout did not match that of the concrete pedestal. In addition, cracks not found in the grout appeared on the concrete pedestal. This result is attributed to the level arm caused by grout failure ahead of failure in the concrete. Eligehausen et al. reported that the spalling of thick grout pads in front of the anchor results in bending of the anchor to transfer the shear load^44^. Despite the higher strength of grout than that of concrete, the grout with a relatively small edge distance starts failing first. Fuchs et al. reported that grout failure ahead of any other type of failure reduces the load transfer capacity^5^. Randle explained this complex interaction through the load-bearing behavior of shear dowels^46^. If the grout can be no longer resistant to the load, a level arm is formed, which results in a complex interaction of tension, shear, and bending stresses developed in the anchor, as shown in Fig. 12. Paschen and Schönhoff investigated the effect of secondary overturning moments in the connection and predicted the shear force corresponding to the concrete breakout of a single anchor, as follows^47^:

1 $$ V_{u,c} \left( M \right) = \psi_{M} \psi_{h,V} V_{b} , $$

where *V*_*b*_ is the basic concrete breakout strength in shear of a single anchor; *ψ*_*M*_ is the moment factor to modify shear strength of anchor to a shear load with level arm; and *ψ*_*h,V*_ is the breakout thickness factor used to modify shear strength of anchors located in concrete members, as follows:

2 $${V}_{b} = 0.6{\left(\frac{{l}_{e}}{d}\right)}^{0.2}\sqrt{d}\sqrt{{f}_{ck}}{\left({c}_{a1}\right)}^{1.5},$$

3 $${\psi }_{M} = (110-\mathrm{e})/90\le 1.0,$$

4 $${\psi }_{h,V} = ({1.5\cdot {c}_{a1}/h)}^{0.5}\ge 1.0,$$

where *l*_*e*_ is the embedment depth of anchor socket, mm.; *d* is the diameter of anchor socket, mm.; *f*_*ck*_ is the specific compressive strength of concrete at test day, MPa.; *c*_*a1*_ is the distance from the center of an anchor shaft to the edge of concrete in one direction, mm.; *e* is the distance between shear load and concrete surface, mm.; and *h* is the thickness of member in which an anchor is located, mm.

An increase in the shear strength by considering the shear capacity of grout bedding, which was not previously considered, was calculated according to Eq. (5) using the ACI 318:

5 $$ V_{gb} =_{ } \frac{{A_{Vg} }}{{A_{Vgo} }}\psi_{hg,V} \psi_{edg,V} V_{g} , $$

where *A*_*Vg*_ is the projected grout failure area of anchor, mm^2^; *A*_*Vgo*_ is the projected grout failure area of anchor if not limited by corner influences, spacing, or member thickness, mm^2^; *V*_g_ is the basic grout breakout strength in shear of a single anchor, with a similar formula to *V*_*b*_; *ψ*_*hg,V*_ is the breakout thickness factor used to modify shear strength of anchor located in grout members, with a similar formula to *ψ*_*h,V*_; and *ψ*_*edg,V*_ is the breakout edge effect factor used to modify shear strength of anchor based on proximity to edges of grout member, *ψ*_*edg,V*_ = 1 is applied in this research.

Thus, the grout effect of the bridge bearing anchor in shear can be obtained as by subtracting the level arm effect created by grout failure from shear strength of anchor embedded in the grout, as follows:

6 $$ Grout\,effect\,in\,shear = V_{gb} - \frac{{A_{Vc} }}{{A_{Vco} }}\psi_{ed,V} V_{u,c} \left( M \right), $$

where *A*_*Vc*_ is the projected concrete failure area of anchor, mm^2^; *A*_*Vco*_ is the projected concrete failure area of anchor if not limited by corner influences, spacing, or member thickness, mm^2^; *ψ*_*ed,V*_ is the breakout edge effect factor used to modify shear strength of anchor based on proximity to edges of concrete member, if *c*_*a2*_ $<$ 1.5*c*_*a1*_*,* then *ψ*_*ed,V*_ = 0.7 + 0.3*c*_*a2*_/(1.5 *c*_*a1*_), otherwise *ψ*_*ed,V*_ = 1.

**Figure 13:**
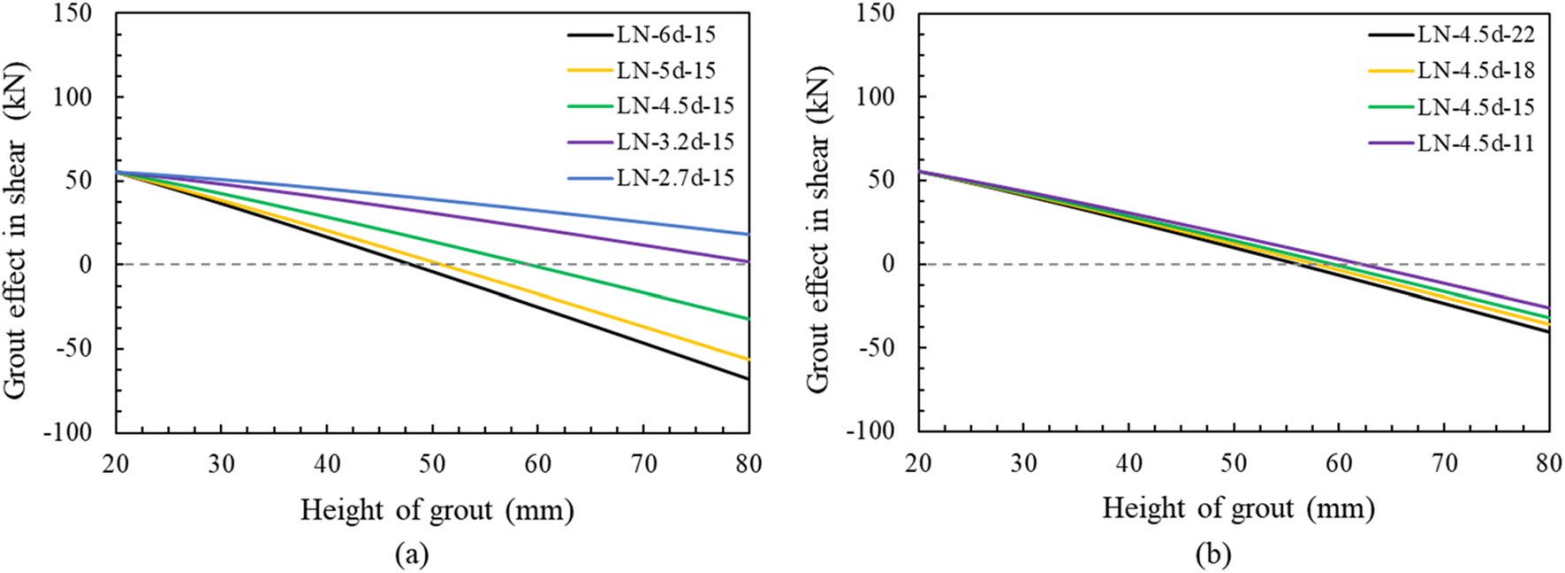
Grout effect in shear with different height of grout: (**a**) edge distance, (**b**) embedment depth.

Figure 13 shows the calculated grout effect in shear resistance according to the grout height in the series of edge distance and embedment depth respectively, which are obtained by Eq. (6). If the grout effect in shear is less than 0, it means that the shear resistance reduction due to the level arm is greater than the improvement in shear capacity caused by grout bedding. As the grout height increases, the grout effect in shear decreases regardless of the edge distance and embedment depth. This result is consistent with many guidelines limiting the grout height due to the stress imbalance in high grout bedding^22,31,43,48^. As the edge distance increased, the grout effect in shear decreased significantly compared to the small decrease shown with embedment depth increase. This is because the grout having a constant edge distance was destroyed first, forming a level arm ahead of concrete failure. The calculation results were similar to the experimental results. While the LN-5d-15 specimen showed a lower maximum shear load than the strength calculated by equation in ACI 318, the specimens with an edge distance less than 5d exhibited the opposite results because these specimens have a positive grout in shear at 50 mm height of grout, as shown in Table 3 and Fig. 13. The grout effect in shear also converged to zero at a height of approximately 50 mm according to the increase in edge distance, and therefore, it was considered appropriate to set the grout height below 50 mm to avoid the negative effect of placing grout bedding on the concrete pedestal.

Furthermore, specimens with a relatively short edge distance of less than 4.5d showed progress on the same inclined failure surface on the grout and concrete pedestal (Figs. A.11, A.12, A.13, A.15). This is because the concrete and grout begin to fail simultaneously before the additional moment is formed by the level arm. Many experiments have conducted without considering the level arm for grout failure^49–51^. Mohamed et al. considered grout properties on shear strength in column base connections and reported that the formation of grout struts plays a major role in the ultimate strength of the connection even beyond the elastic range zone^52^. A grout bedding is designed to have a shorter edge distance than that of concrete in bridge bearing anchor. The current design code does not reflect the effects of grout; however, it should be considered to limit the height of grout bedding to minimize this negative effect in terms of overall stability in shear.

#### Displacement behavior of grout bedding and concrete pedestal

Inspection and maintenance of bridge bearing anchor are generally performed on visible destructions, such as failure of the anchor bolt, concrete pedestal, and large cracks both in grout bedding and concrete pedestal^53^. However, it is not easy to identify internal cracks that do not extend to the free surface under certain loading, because the concrete around the anchor is located under the grout bedding. These small internal cracks in the concrete may not yet have a significant effect on structural performance; however, they might be vulnerable to chemical penetration, such as de-icing materials coming down from the bridge girder by affecting the durability of concrete^54^. ACI 224R-01 recommends a maximum allowable crack width of 0.18 mm for concrete exposed to de-icing chemicals^55^. Meanwhile, non-destructive testing (NDT) of concrete cracks is normally used for the maintenance of these concrete structures, and numerous methods are changing with the development of detection devices^56^. However, Titman emphasized the need for care and experience despite the advantages of the NDT methods, and indicated that inaccurate interpretation can be made in the absence of a specialized technique^57^.

**Figure 14:**
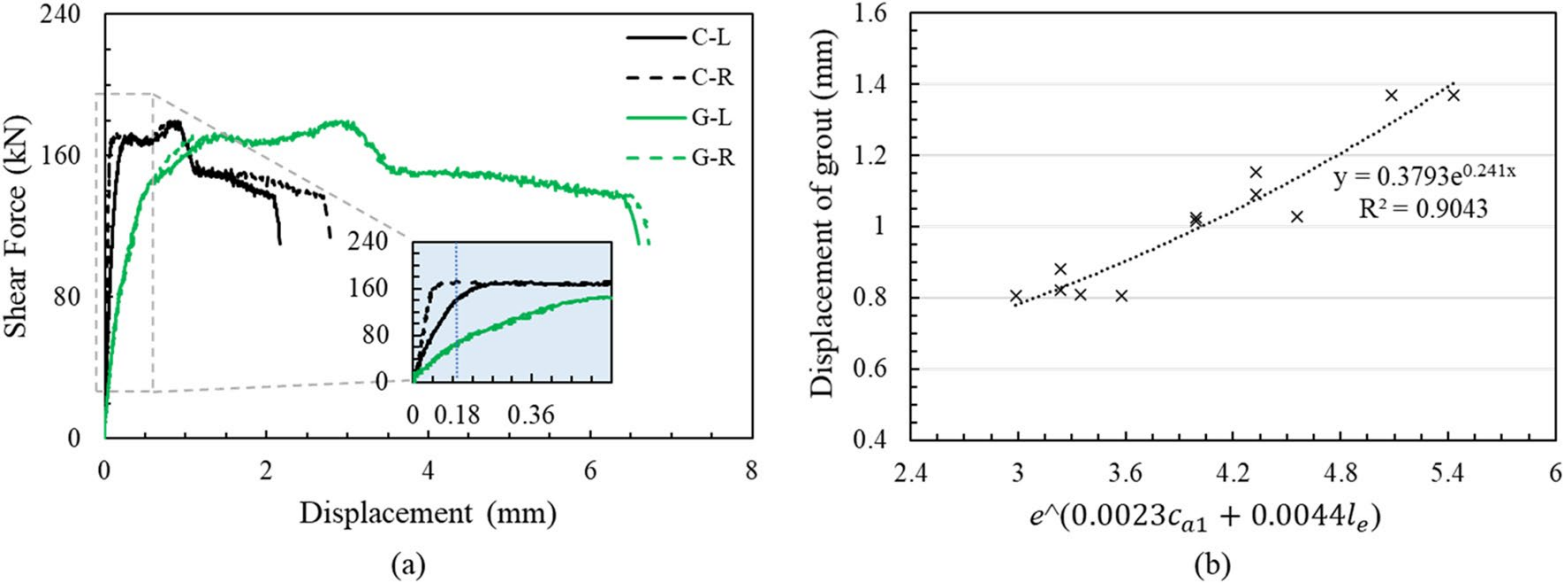
Displacement behavior of grout bedding and concrete pedestal: (**a**) load–displacement curve of LN-3.2d-15 specimen for grout and concrete, (**b**) displacement of grout due to edge distance and embedment depth.

As previously described, cracks occur in the grout first and then stress redistribution occurs in the anchor, resulting in cracks in the concrete. A simple initial inspection is possible if the crack width of the concrete is relatively predictable through the grout crack. In other words, the degree of cracking of the concrete underneath can be inferred from the degree of cracking of the grout. In this study, the concrete was completely fixed by applying hydraulic pressure and no movement of the substructure occurred, as measured by LVDT. The displacements of the left and right points 100 mm away from the predicted failure line of the front center part of both the concrete pedestal and grout bedding were measured using LVDTs. As an example, the load displacement curve of the LN-3.2d-15 specimen for the grout and concrete pedestal is shown in Fig. 14a. Here, C-L and C-R represent the values of the left and right points of the concrete pedestal, respectively, in the same manner in which G-L and G-R correspond to grout bedding. As the load increased, the displacement of the grout increased rapidly compared to the displacement of the concrete. The maximum displacement of the grout was also higher than that of the concrete pedestal. The average grout displacement of left and right points was obtained at the same load when the concrete displacement was 0.18 mm. The displacements of grout for specimens with a low concrete pedestal with normal strength were summarized according to the edge distance and embedment depth. As a result of the regression analysis, the grout displacements was expressed as an exponential function according to the edge distance and embedment depth with R^2^ = 0.97 and 0.91, respectively. Therefore, it can be represented as Fig. 14b if the two variables are combined by setting the product of the two exponential functions as the x-axis to extend the applicability. The degree of crack width of the concrete could be predicted relatively through the grout displacement by inputting the designed edge distance and embedment depth of the bridge bearing anchor with a normal-strength concrete pedestal through the curve.

### Shear resistance of anchor reinforcement

Few studies have been conducted on anchors reinforced by a straight reinforcement commonly used in construction, while there are some experimental studies on the hairpin^58^. A bridge bearing anchor adopts the method of stirrup reinforcement, which extends to the substructure reinforcement. The ACI 318 simply increases the strength through a breakout cracking factor *ψ*_*c,V*_ for an anchor with reinforcement based on the breakout strength, rather than considering the resistance by the reinforcement^2,38^. Whereas, EN 1992-4 considers the effect of supplementary reinforcement in the form of stirrups and edge reinforcement on shear performance by presenting equation^3^. Eligehausen et al. reported that a narrow stirrup spacing with large edge distance increases the restraint effect and evaluated the resistance strength of the stirrup reinforcement by applying the concept of the strut-tie model^44^. Sharma et al. developed a model for single and multiple row anchorage with supplementary reinforcement by improving the formula proposed by Schmid^59–62^.

**Figure 15:**
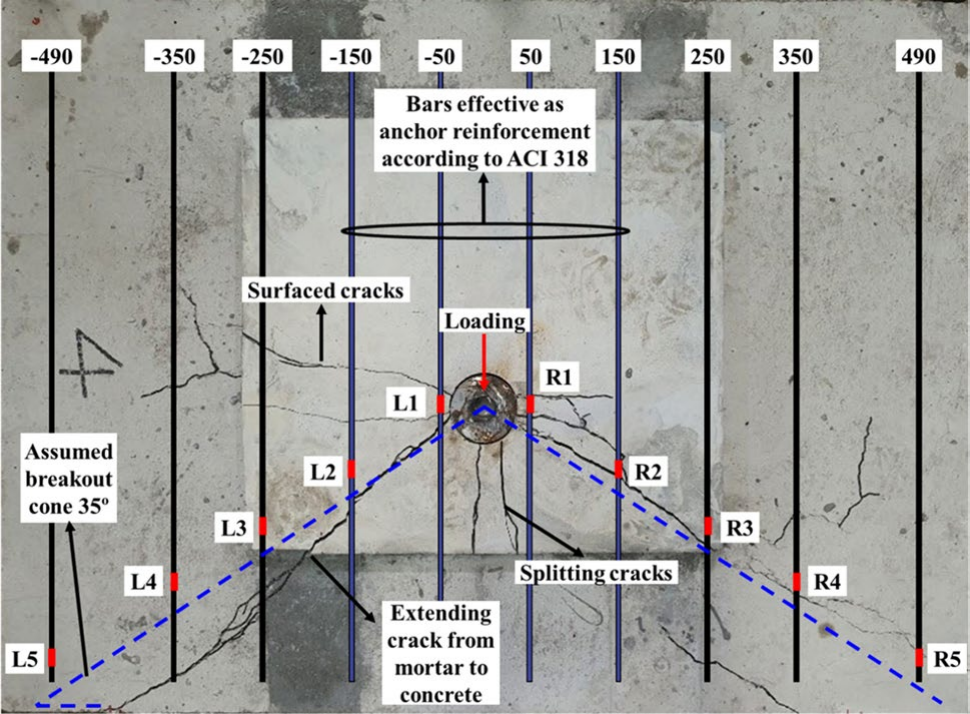
Locations of stirrup and strain gauge (LN-5d-15).

**Figure 16:**
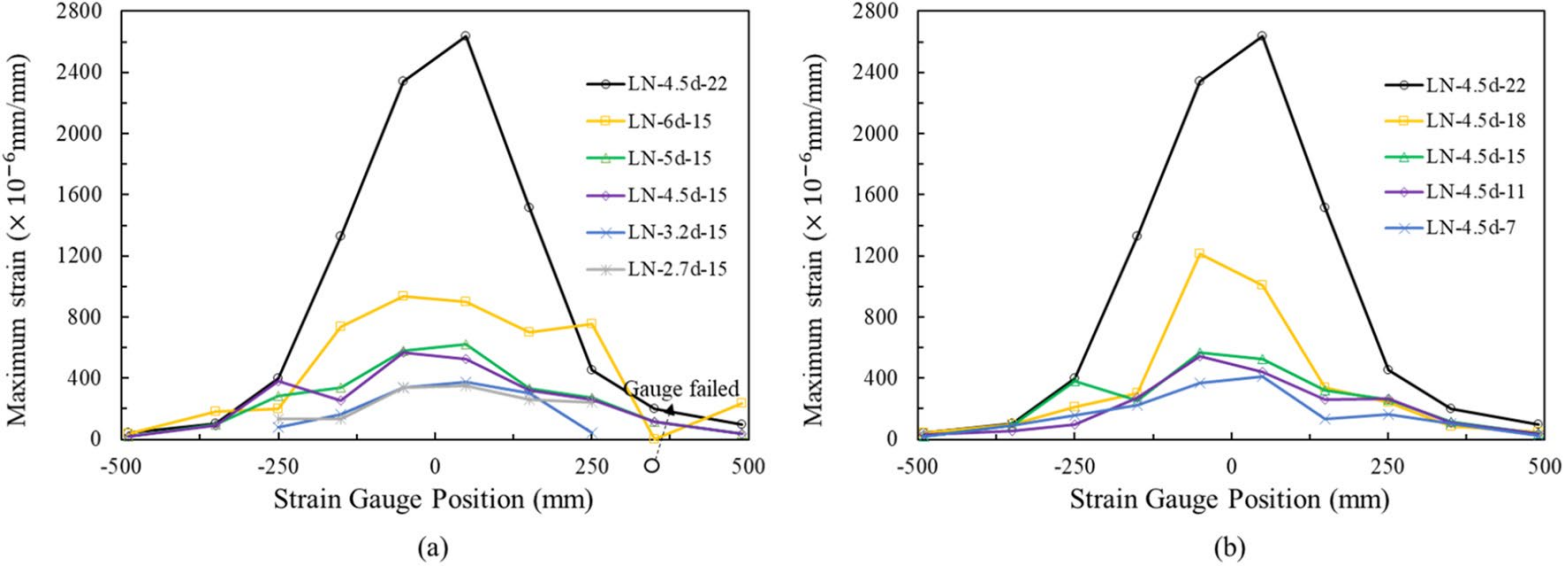
Maximum strain in anchor stirrup reinforcement: (**a**) edge distance, (**b**) embedment depth.

The details of the stirrup reinforcement and strain gauge locations are shown in Fig. 15. The strain gauges were installed 25 mm behind the assumed breakout surface line on the surface reinforcements. The effective anchor reinforcements proposed by the ACI 318 are stirrups that fall within 0.5 ${c}_{a1}$ or 0.3 ${c}_{a1}$ from the anchor, whereas stirrups within a distance of 0.75 ${c}_{a1}$ are considered effective by EN 1992-4. The LN-5d-15 specimen corresponded to four stirrups, whereas the specimens with edge distance less than 4.5d corresponded to only two stirrups according to ACI 318. Larger strains were generally observed in the stirrup closer to the anchor, as shown in Fig. 16. Only the LN-4.5d-22 specimen showed the strain of the stirrup next to the anchor exceeded the yield strain. The resistance by stirrups was found to be more dominant by embedment depth than edge distance. The influence of the surface reinforcement may be greater than that of the reinforcement in the substructure because the breakout cone originates at the top of the embedment depth. However, if the anchor is embedded up to the reinforcement in the substructure, it bears edge reinforcement both of the surface and substructure. Thus, the constraining effect of the strut-tie through concrete and reinforcements can be enhanced, resulting in an increase in shear resistance.

**Figure 17:**
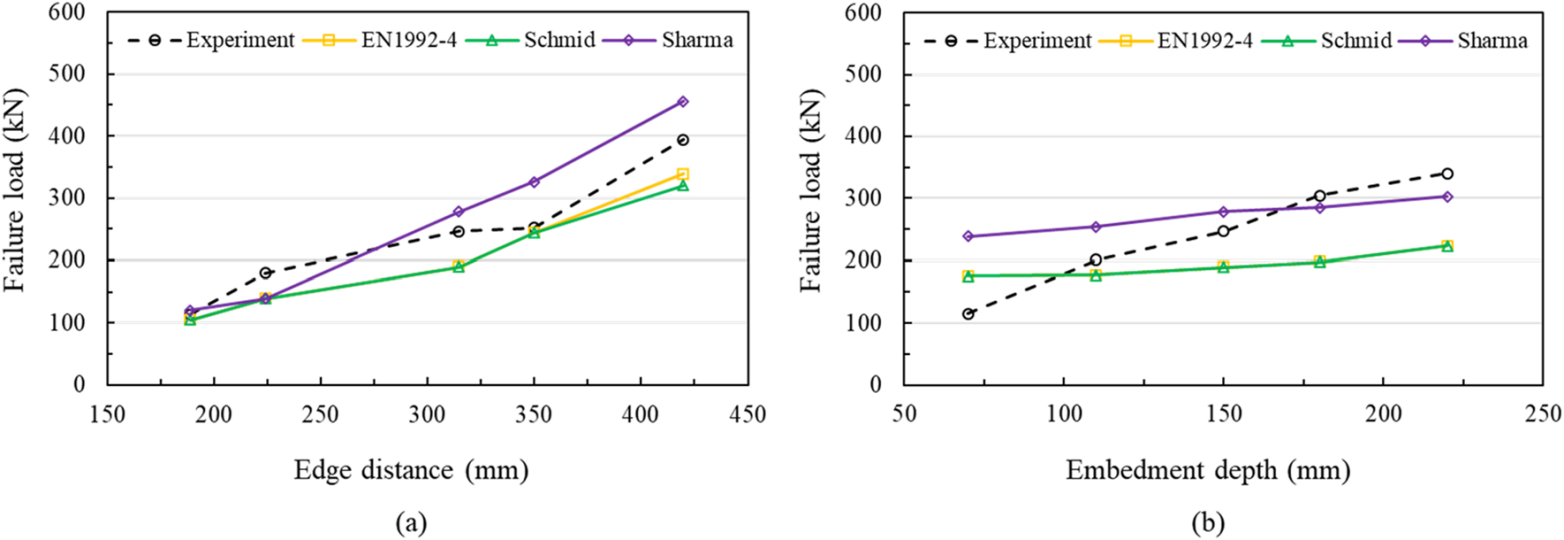
Comparison of failure loads obtained from the tests with calculated failure loads through EN1992-4^3^, Schmid^59^, and Sharma^62^: (**a**) effect of the edge distance at an embedment depth of 150 mm, (**b**) effect of the embedment depth at an edge distance of 4.5d.

Figure 17 represents comparison of experimentally obtained and analytically evaluated failure loads by calculations according to EN 1992-4, Schmid model and Sharma model, which consider the effect of supplementary reinforcement at formula. The resistance obtained from EN 1992-4 and Schmid model represented similar failure loads because the calculated mean concrete edge breakout resistance of anchorage was greater than calculated mean resistance of the supplementary reinforcement of anchorage failure in the concrete edge breakout body in most specimens. The failure loads from EN 1992-4 and Schmid model showed the conservative results compared to experimental results expect for the specimen with an embedment depth of 70 mm. The specimens with shallow embedment depth were not fully embedded, thus actual contribution of shear resistance by reinforcement were insignificant as explained earlier (Fig. 16). This results represented the difficult in securing the safety of the bridge bearing anchor system with shallow embedment depth. The failure loads from the Sharma model represented conservative results when the edge distance decreased by 3.2d and the embedment depth increased by 180 mm, as shown in Fig. 17a,b, respectively. The overall curve progression of the experiments was followed nicely by the Sharma model. Lever arm in the bridge bearing anchor could be a quite high compared with other anchor system. If the anchor is fully embedded, the support reinforcement may yield and represent conservative design value (Fig. 17b), otherwise, the effective length of the bar may decrease along with the level arm effect, indicating a non-conservative results. Therefore, it is considered that the failure loads of specimen with large edge distance can be more similar to the experimental results if the embedment depth is fully secured.

### Design recommendations

#### Anchor sockets in bridge bearing anchor

Anchor sockets are used for the convenience of construction in bridge bearing anchors. In the current design codes, diameter of anchor socket is simply considered as diameter of anchor. However, the behavior of anchor socket is quite different from general anchors, which fail as a result of anchor shaft fracture without any bending^50^. In the bridge bearing anchor, the upper part of the anchor bolt in the load plate resists the shear load first, and then the load is transmitted to the concrete through the anchor socket combined with the anchor bolt. Because the diameter of the socket is relatively larger than that of the anchor bolt, ductile behavior of the anchor socket cannot be expected in the shear load. Therefore, brittle fracture occurs due to stress concentration at the contact point between the socket top and the bolt, which is not directly embedded in the concrete. It can be confirmed that the bolts were broken and bent at the contact point, as shown in Fig. 8. The anchor bolt suddenly failed after the load decreases at different peak loads, which is not as expected. In addition, anchor sockets are mostly round form, while general anchors have deformed lines in the shaft. In most specimens, such as LN-4.5d-18 and LN-3.2d-15, bond failure between the anchor socket and grout occurred, which finally showed breakout failure. There is much need to improve the bond between grout and anchor socket, also between concrete and anchor socket. Thus, deformed anchor sockets need to be used and should be verified experimentally to prevent bond failure for full breakout strength, as indicated by Chicchi et al.^63^.

#### Breakout strength of bridge bearing anchor with low concrete pedestal

A bridge bearing anchor is generally composed of anchor groups, whose number is determined according to the bearing type, and mostly comprises four anchors. The anchor should be designed based on the steel strength of the anchor, breakout strength, and pryout strength. Because anchor bolts are determined according to the bearing type, both the general diameter and strength have already been generalized. The pryout strength of a general anchor embedded up to the substructure reinforcement is generally higher than the breakout strength in shear^43,64^. Therefore, the analysis of the design formula for the breakout strength was confirmed based on the results.

Multiple nonlinear regression analysis was performed on variables of low concrete pedestal to avoid considering the effects on the breakout thickness factor *ψ*_*h,V*_ and projected concrete failure area *A*_*Vc*_ among the variables failed by breakout failure. The concrete breakout strength was shown to be almost proportional to the square root of the compressive strength, as previously described. Therefore, the compressive strength was not considered as a variable in the regression analysis. A regression analysis with two independent variables, which are edge distance and embedment depth, was performed. The breakout strength of the bridge bearing anchor can be expressed as follows:

7 $${V}_{pb}=1.065{\left(\frac{{l}_{e}}{d}\right)}^{0.644}\sqrt{d}\sqrt{{f}_{ck}}{\left({c}_{a1}\right)}^{1.421}\left({\mathrm{R}}^{2} = 0.98\right).$$

**Figure 18:**
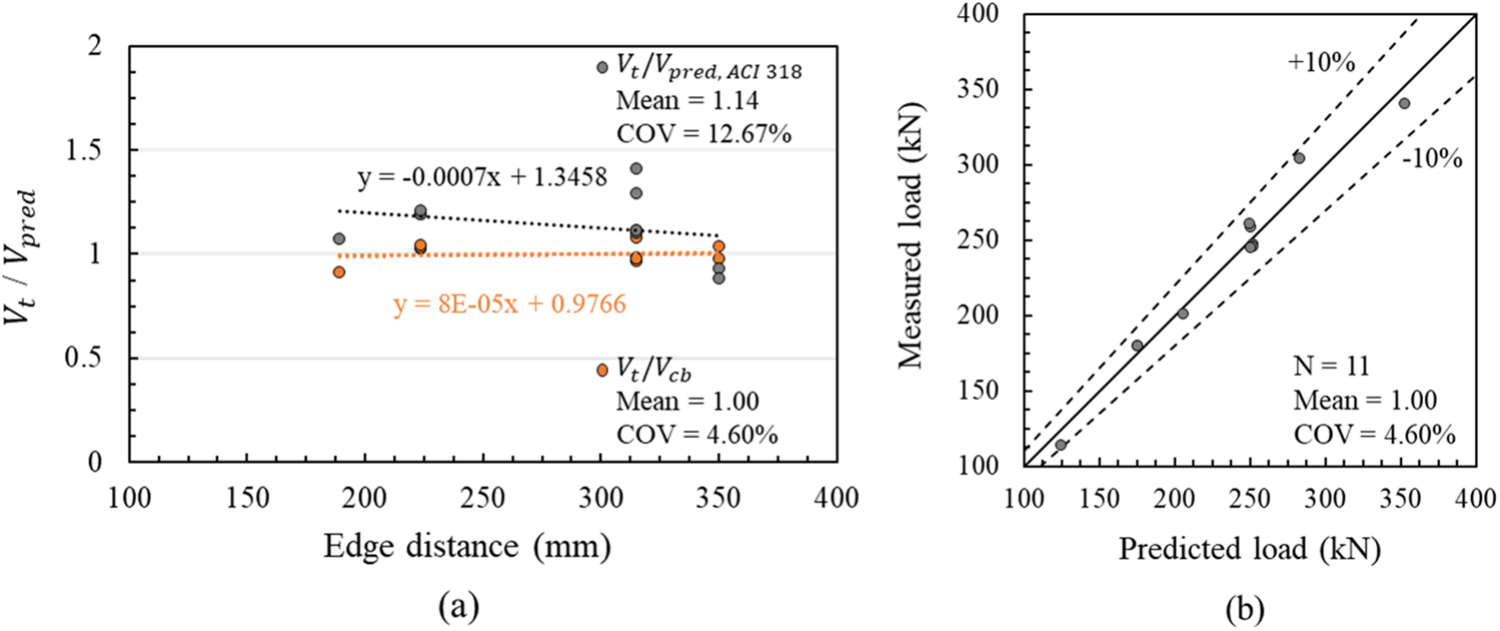
Comparison between predicted and measured loads: (**a**) edge distance, (**b**) predicted load by Eq. (4).

The derived design equation showed that the power of $({l}_{e}/d)$ increased significantly compared to that indicated in the ACI 318, which adopted ${\left({l}_{e}/d\right)}^{0.2}$. This corresponds to the previous results affected by the reinforcement of the substructure depending on the embedment depth. The bearing stress distribution along the load transfer anchor shaft was also highly influenced by restriction through substructure reinforcement^65^. The power of ${c}_{a1}$ decreased a little compared to that indicated in the ACI 318, which adopted ${{c}_{a1}}^{1.5}$. This result agrees with those of the comparison test result and predicted strength due to the edge distance, as shown in Fig. 18a. The negative slope of the comparison indicates that the design formula of the ACI 318 accounts for the influence of the edge distance excessively in the bridge bearing anchor^66^. As the edge distance increases, the shear capacity may decrease because of the effect of the level arm. A comparison with the load predicted by Eq. (7) and the measured load showed only 4.60% of COV (coefficient of variation) and a difference of less than 10%, as shown in Fig. 18b.

For simplicity of the design equation, Eq. (7) can be modified to Eq. (8) with powers of 2/3 and 1.4 for $\left({l}_{e}/d\right)$ and ${c}_{a1}$ respectively, which shows 4.63% of COV:

8 $${V}_{bp}=1.18{\left(\frac{{l}_{e}}{d}\right)}^\frac{2}{3}\sqrt{\mathrm{d}}\sqrt{{f}_{ck}}{\left({c}_{a1}\right)}^{1.4}\left({\mathrm{R}}^{2} = 0.98\right).$$

The ACI 318 for the strengths of anchors adopts the 5% fractile concept for the safety of the structure. The concept of 5% fractile indicates a 90% confidence that the actual strength exceeds the nominal strength with 95% probability^2,67,68^. The coefficient of 5% fractile, *F*_*5%*_, was calculated as 0.884 using the mean, COV, and number of specimens. Consequently, the nominal concrete breakout strength of the bridge bearing anchor in shear can be obtained as follows^18^:

9 $${V}_{b, bearing}= \frac{{F}_{5\%}}{{\psi }_{c,V}}{V}_{pb}=0.75{\left(\frac{{l}_{e}}{\mathrm{d}}\right)}^{2/3}\sqrt{\mathrm{d}}\sqrt{{f}_{ck}}{\left({c}_{a1}\right)}^{1.4}.$$

The breakout strength ${V}_{cb}$ can be calculated by considering the coefficient of the bridge bearing anchor, such as the effective area of the group anchors, replacing ${V}_{b}$ of the ACI 318 with ${V}_{b,bearing}$. This equation is only applicable to this research stage because the major factors such as height and strength of grout bedding and different types of supplementary reinforcement are not considered here. Therefore, more research is need to solidify its applicability for all bridge bearing anchor cases.

## Conclusions

This research investigated the shear capacities of cast-in-place single anchors simulating the characteristics of bridge bearing anchor under quasi-static loads. Twenty-one anchor specimens with different edge distance, embedment depth, and compressive strength of the concrete were fabricated and tested. Based on the results and analysis detailed above, the following conclusions were drawn:

1. Three types of failure modes were observed according to the edge distance, embedment depth, and compressive strength. If the breakout strength was much greater than the steel strength of the anchor, the bolt showed brittle failure under load higher than the predicted strength. In case of a short embedment depth, such as the LN-4.5d-7 specimen, failure load occurred near the pryout strength; however, no large spalling was observed on the rear part of the anchor due to the bearing effect of the reinforcement.
2. The shear capacities of the anchors were different from the strengths obtained by design equation in the ACI 318 and EN 1992-4; in particular, the embedment depth had a greater influence on shear resistance than that considered in the current codes due to the existence of both surface and substructure reinforcements. As the height of the concrete pedestal increased, the stress distribution from the bearing to the substructure was not well-transmitted, especially with shallow embedment depth specimens.
3. The grout bedding on the concrete pedestal was locally damaged before serious damage was caused to the concrete, forming a level arm, which induced a decrease in shear capacity. The effect of grout on shear resistance increased as the grout height decreased, and was influenced by the edge distance rather than the embedment depth. Through the analysis of displacement behavior of grout bedding and concrete pedestal, an equation of curve was proposed to predict the relative cracking degree of the concrete under the grout.
4. The strain of the surface stirrup reinforcement was measured, and a high strain occurred in the rebar close to the anchor. The behavior of the strain was more influenced by the embedment depth than the edge distance; thus, increasing the embedment depth up to the substructure reinforcement was found to be effective in improving the resistance of the stirrups against shear load.
5. The comparison of experimentally obtained and analytically evaluated failure loads by calculations according to EN 1992-4, Schmid model and Sharma model was conducted to consider the effect of supplementary reinforcement. The failure loads of the bridge bearing anchor were higher than those calculated by the EN 1992-4 and Schmid model except for specimens with embedment depth of 70. The failure loads calculated by the Sharma model were lower than the experimental failure load according to a decrease in edge distance and increase in embedment depth.
6. Multiple nonlinear regression analysis was performed for anchors with low concrete pedestals where breakout failure occurred, and a design equation was derived to enable a more precise prediction of the bridge bearing. The power of compressive strength showed a tendency similar to that of the code, and the powers of the embedment depth and edge distance were modified. The 5% fractile concept was also introduced into the equation to ensure safety of the structure.

## Supplementary Information

Supplementary Information.

## Data Availability

The datasets generated during and analysed during the current study are available from the corresponding author on reasonable request.
