# Supplementary material for: Shear behavior of single cast-in anchor simulating characteristics of bridge bearing anchor
Source: Sci Rep. 2022 Aug 3;12:13308. doi: 10.1038/s41598-022-17027-z (PMC9349318; doi:10.1038/s41598-022-17027-z)
Supplement: Supplementary file 1 — Supplementary Information. [file 41598_2022_17027_MOESM1_ESM.docx]

**Appendix A: Failure Mode**

**Fig. A.1** LN-6d-15

**Fig. A.2** LN-6d-7

**Fig. A.3** LN-5d-15

**Fig. A.4** LN-5d-15 (2)

**Fig. A.5** LN-4.5d-22

**Fig. A.6** LN-4.5d-18

**Fig. A.7** LN-4.5d-15

**Fig. A.8** LN-4.5d-15 (2)

**Fig. A.9** LN-4.5d-11

**Fig. A.10** LN-4.5d-7

**Fig. A.11** LN-3.2d-15

**Fig. A.12** LN-3.2d-15 (2)

**Fig. A.13** LN-2.7d-15

**Fig. A.14** LH-4.5d-15

**Fig. A.15** LH-3.2d-15

**Fig. A.16** HN-6d-15

**Fig. A.17** HN-4.5d-30

**Fig. A.18** HN-4.5d-23

**Fig. A.19** HN-4.5d-15

**Fig. A.20** HN-4.5d-15 (2)

**Fig. A.21** HH-4.5d-15


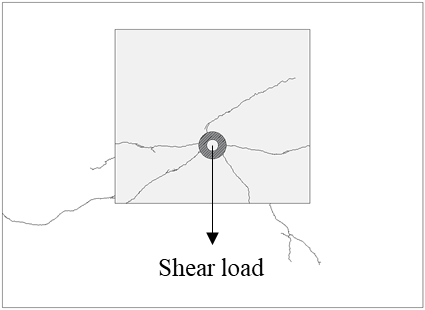

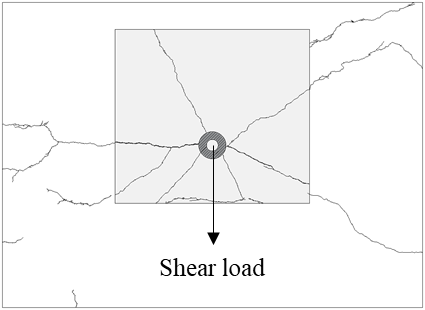


**Fig. A.1** LN-6d-15 **Fig. A.2** LN-6d-7


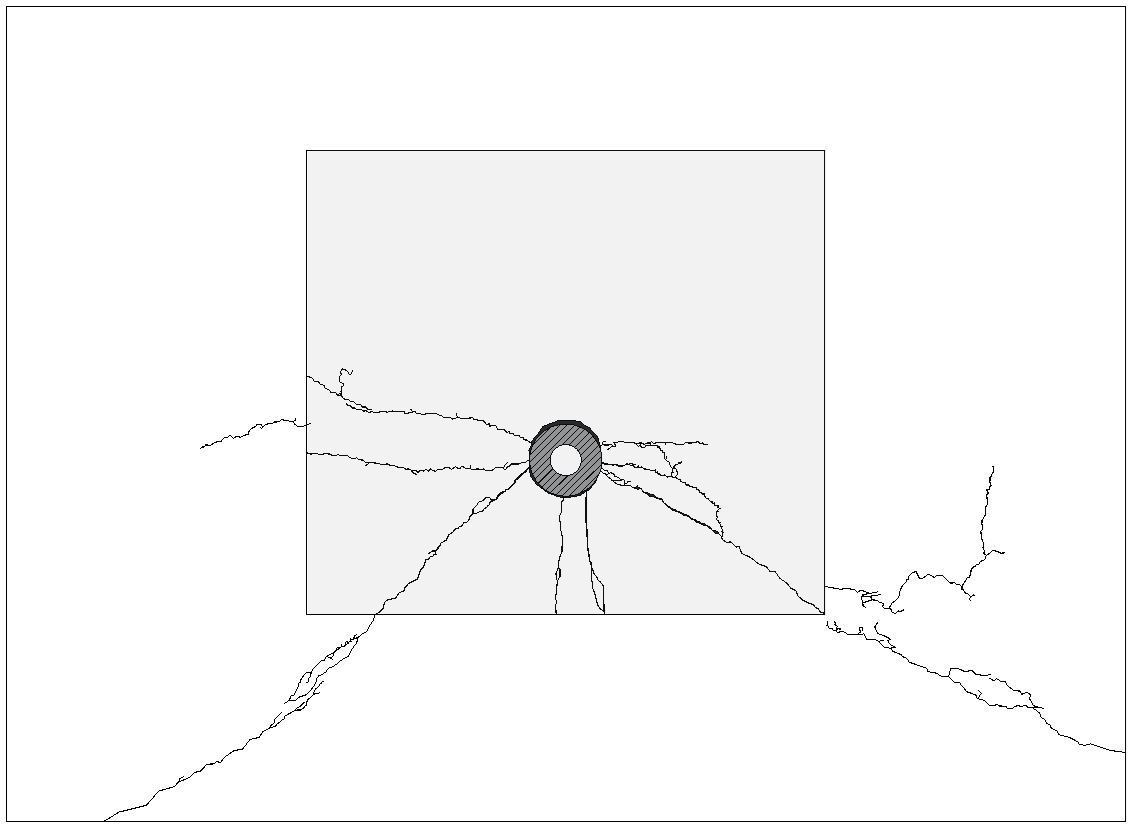

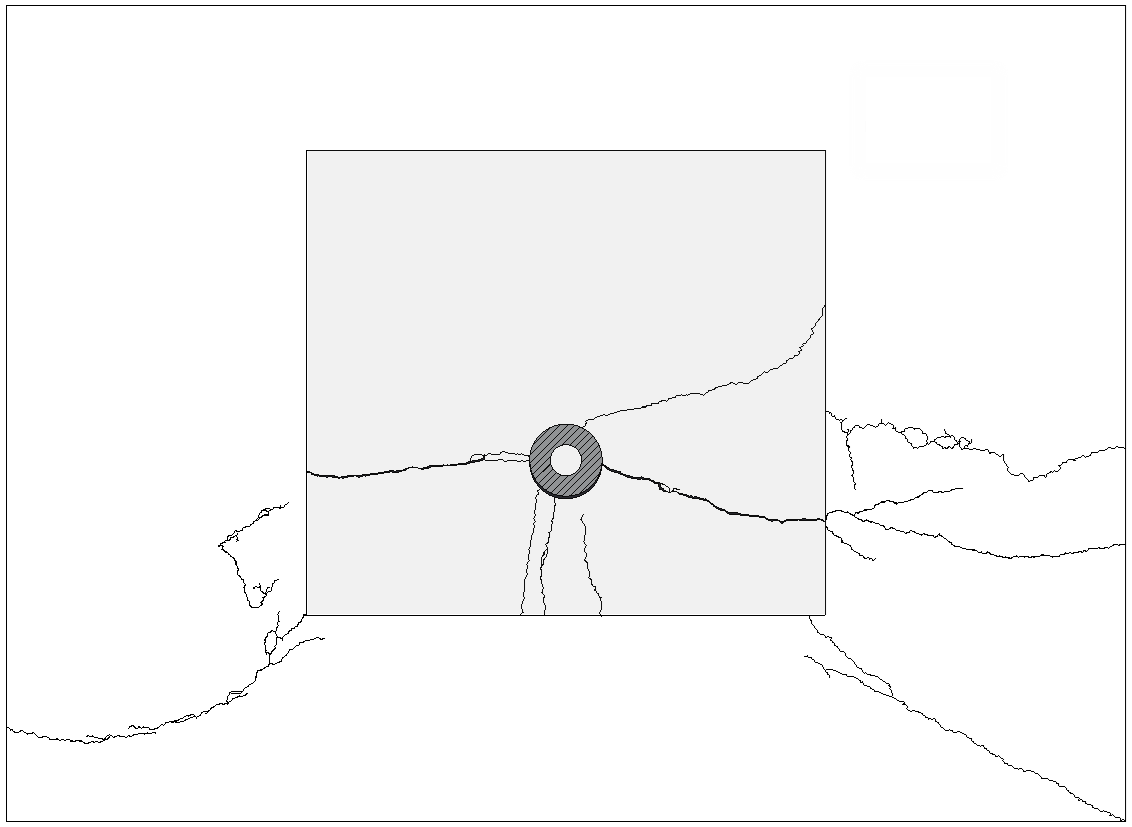


**Fig. A.3** LN-5d-15 **Fig. A.4** LN-5d-15 (2)


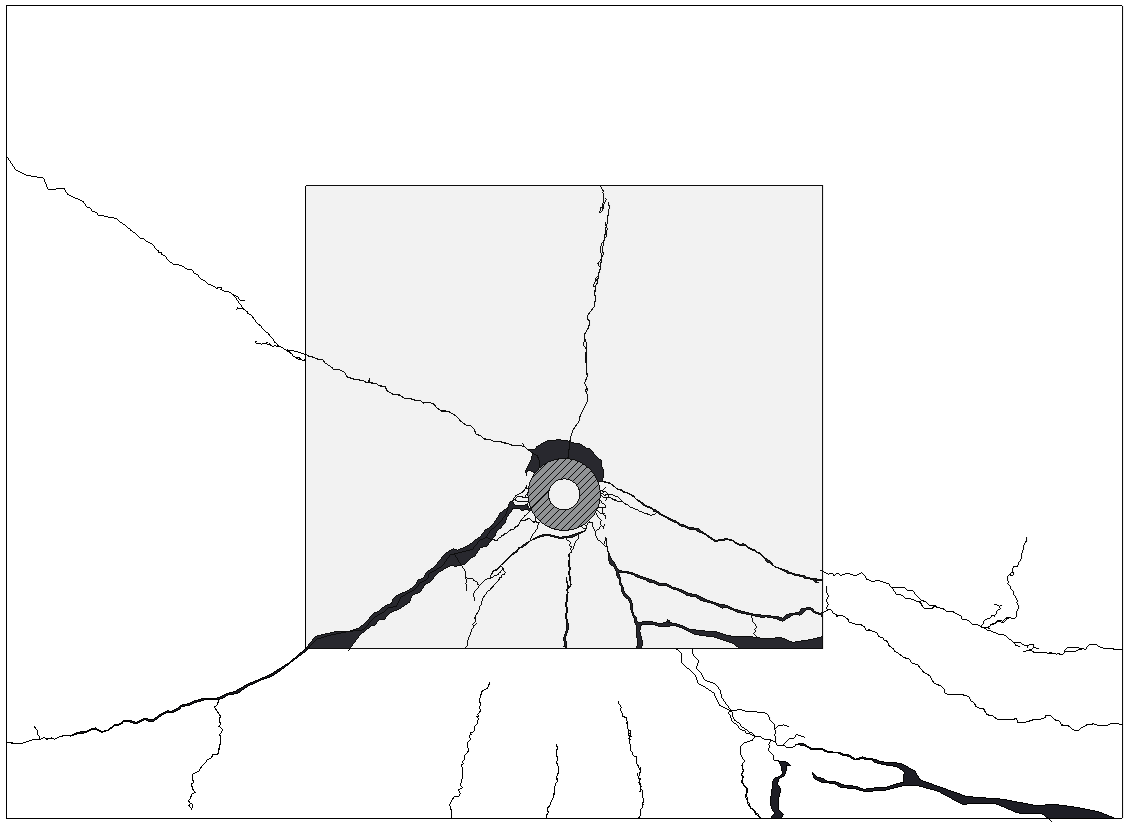

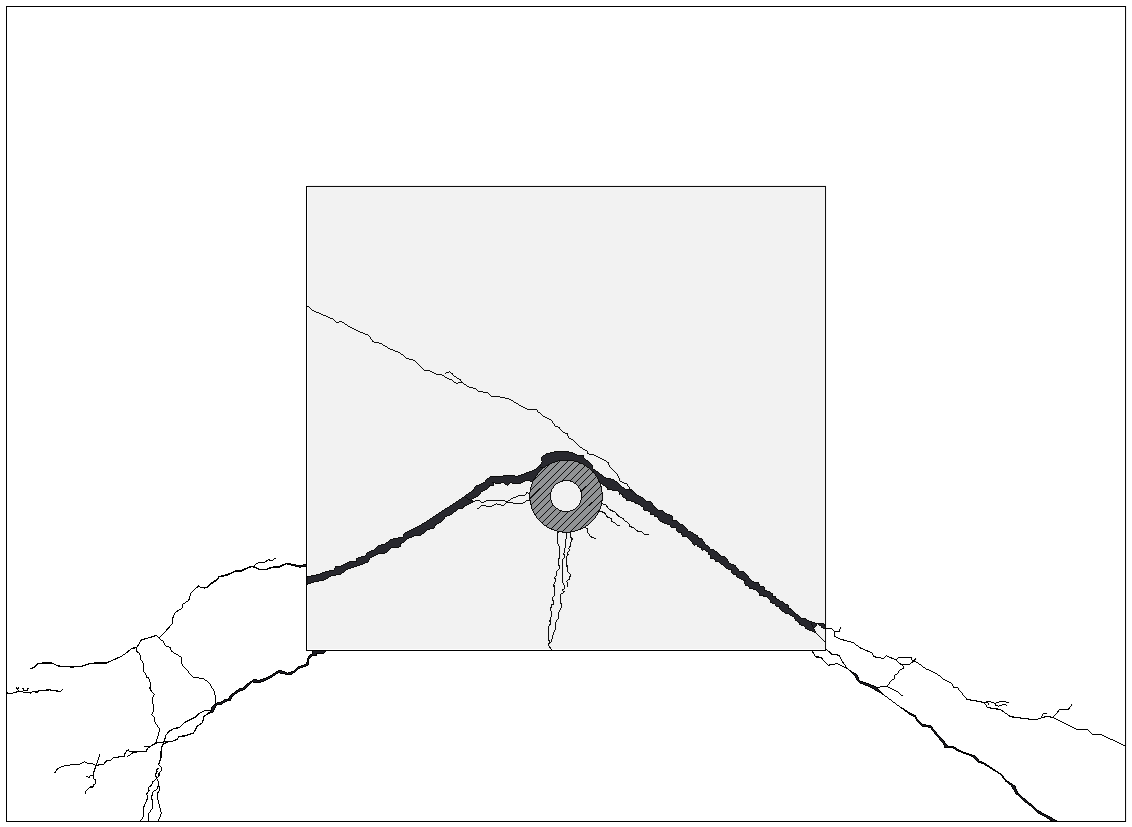


**Fig. A.5** LN-4.5d-22 **Fig. A.6** LN-4.5d-18


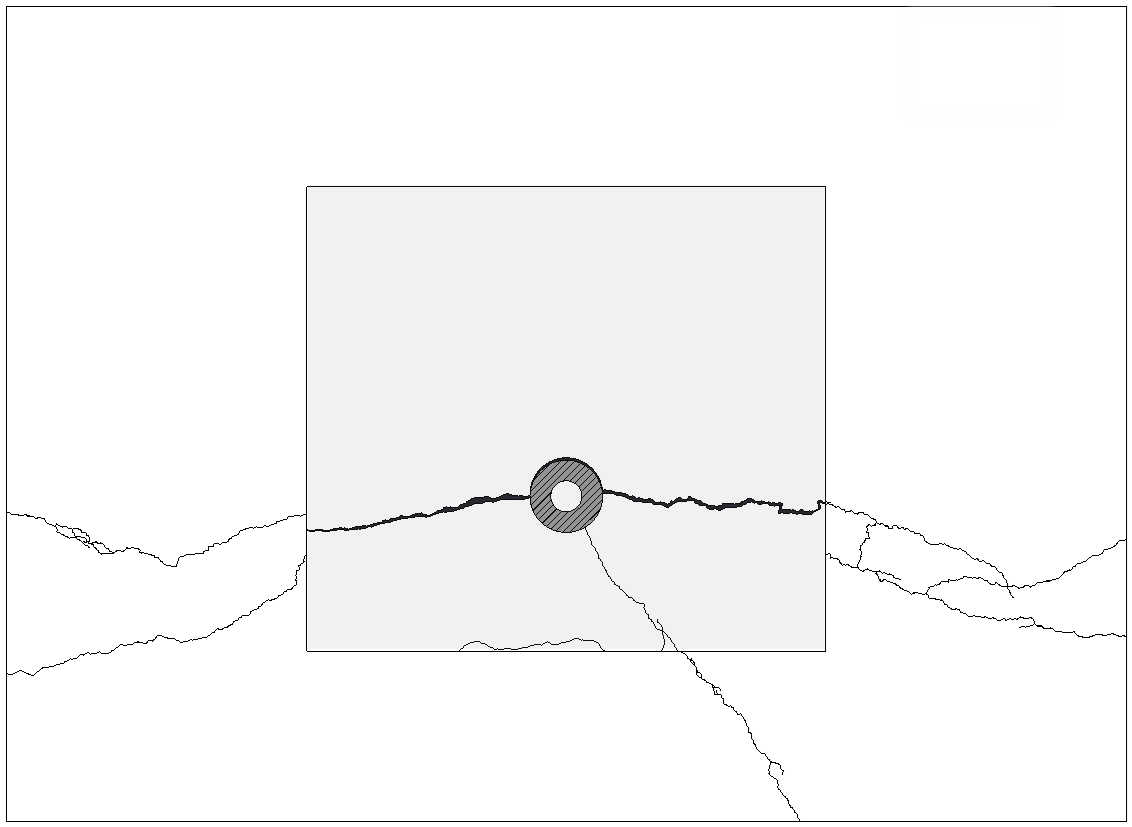

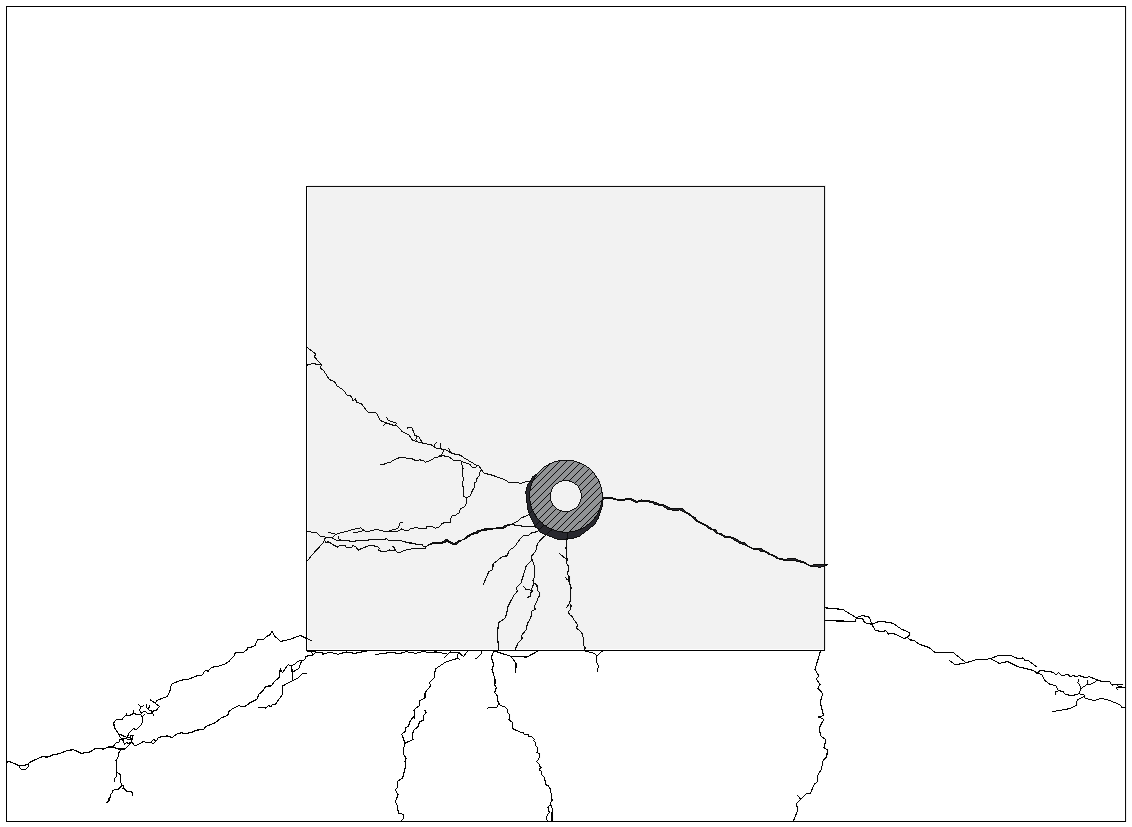


**Fig. A.7** LN-4.5d-15 **Fig. A.8** LN-4.5d-15 (2)


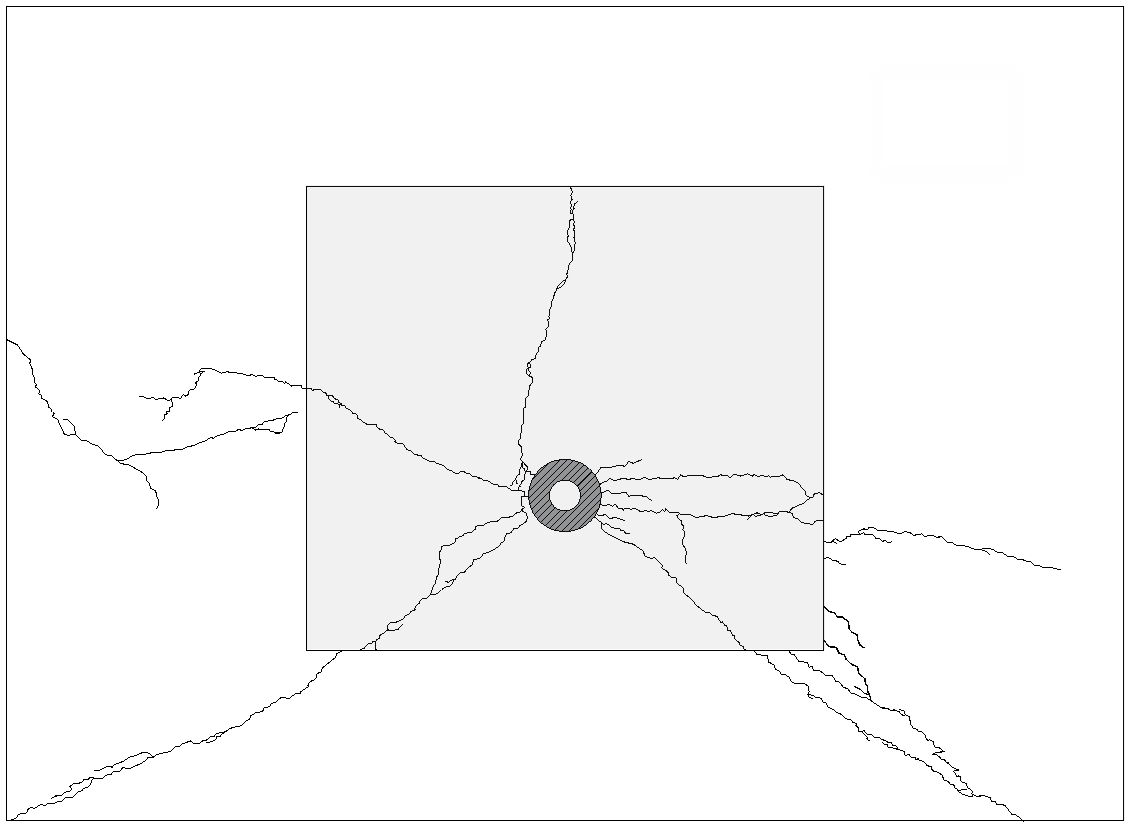

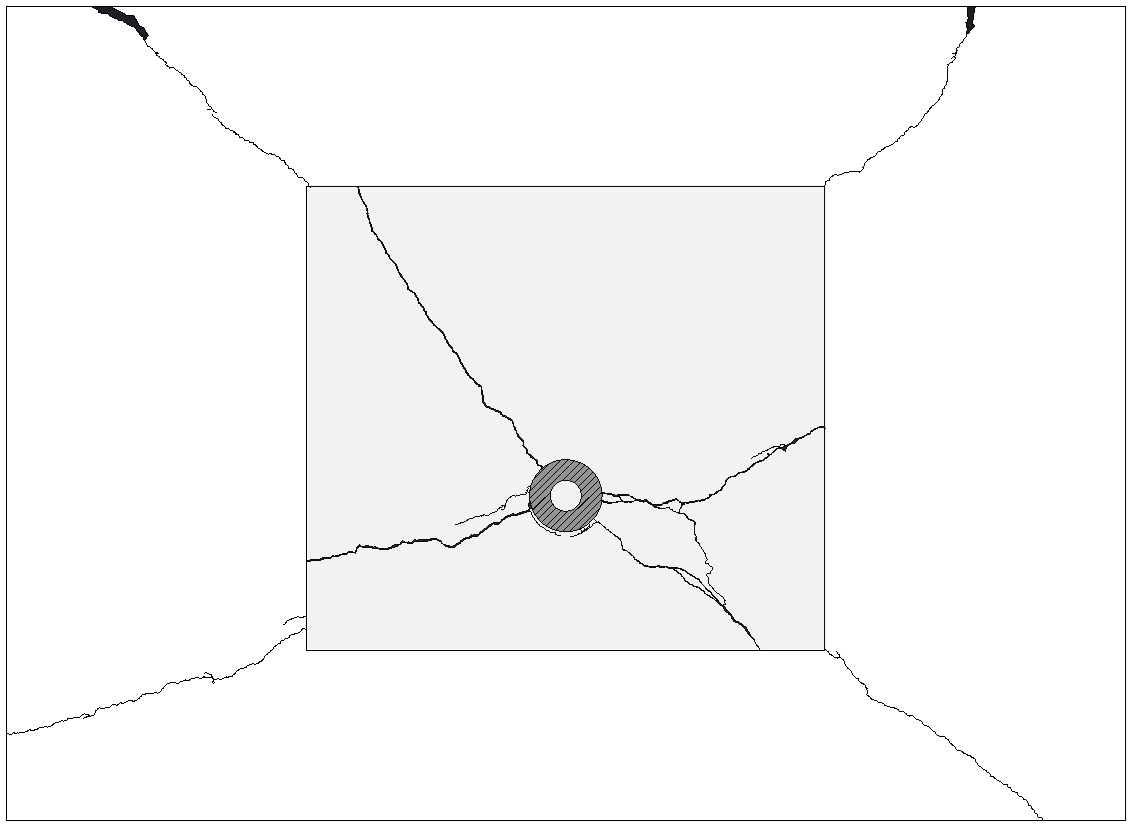


**Fig. A.9** LN-4.5d-11 **Fig. A.10** LN-4.5d-7


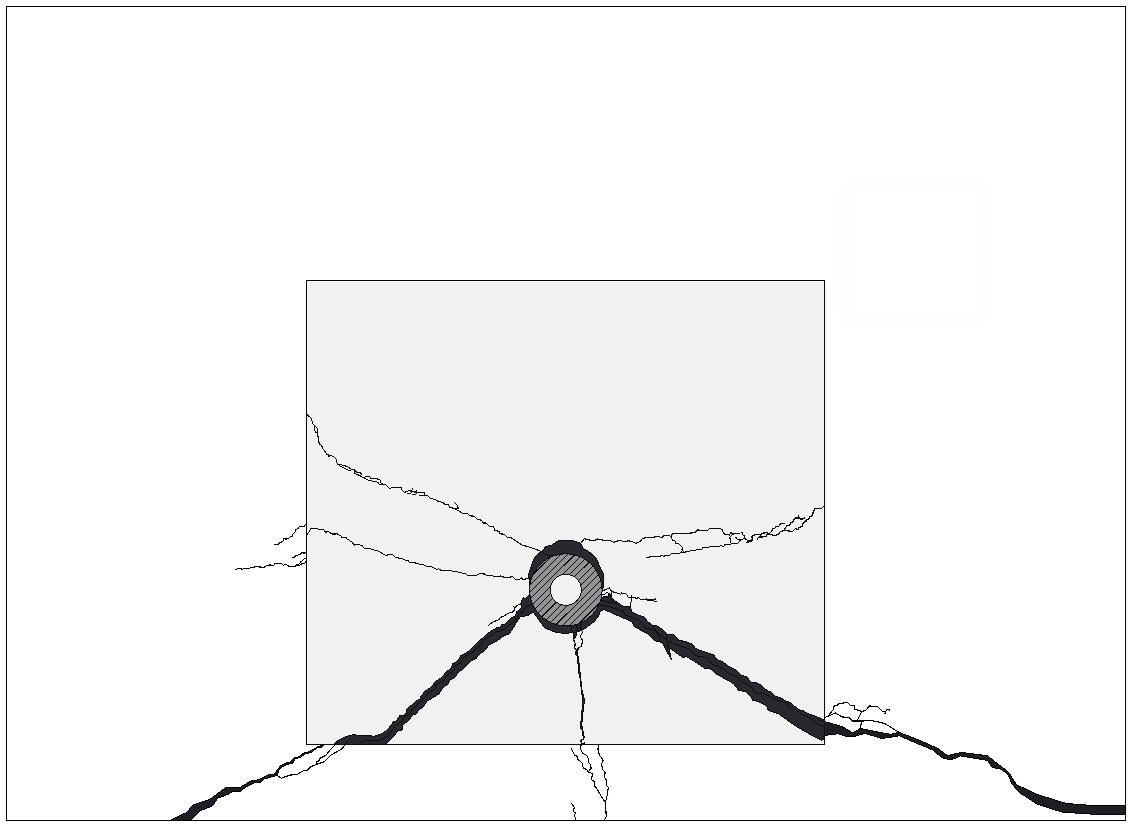

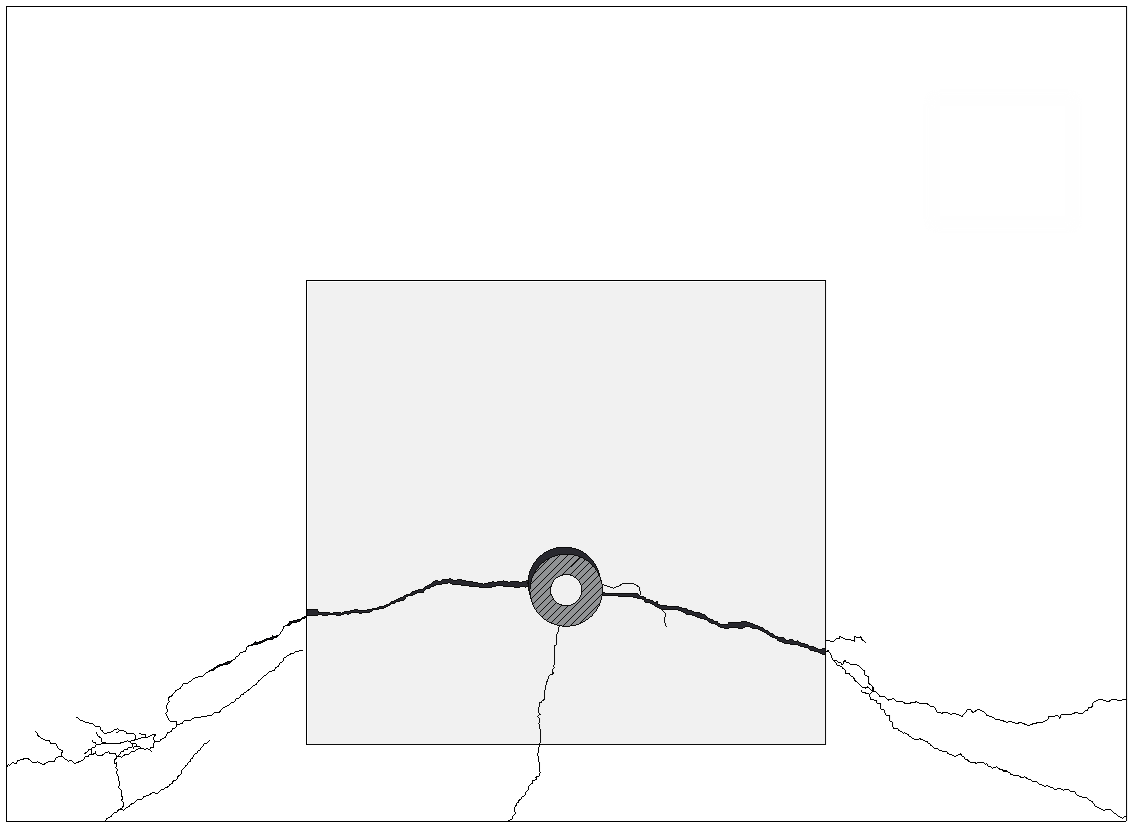


**Fig. A.11** LN-3.2d-15 **Fig. A.12** LN-3.2d-15 (2)


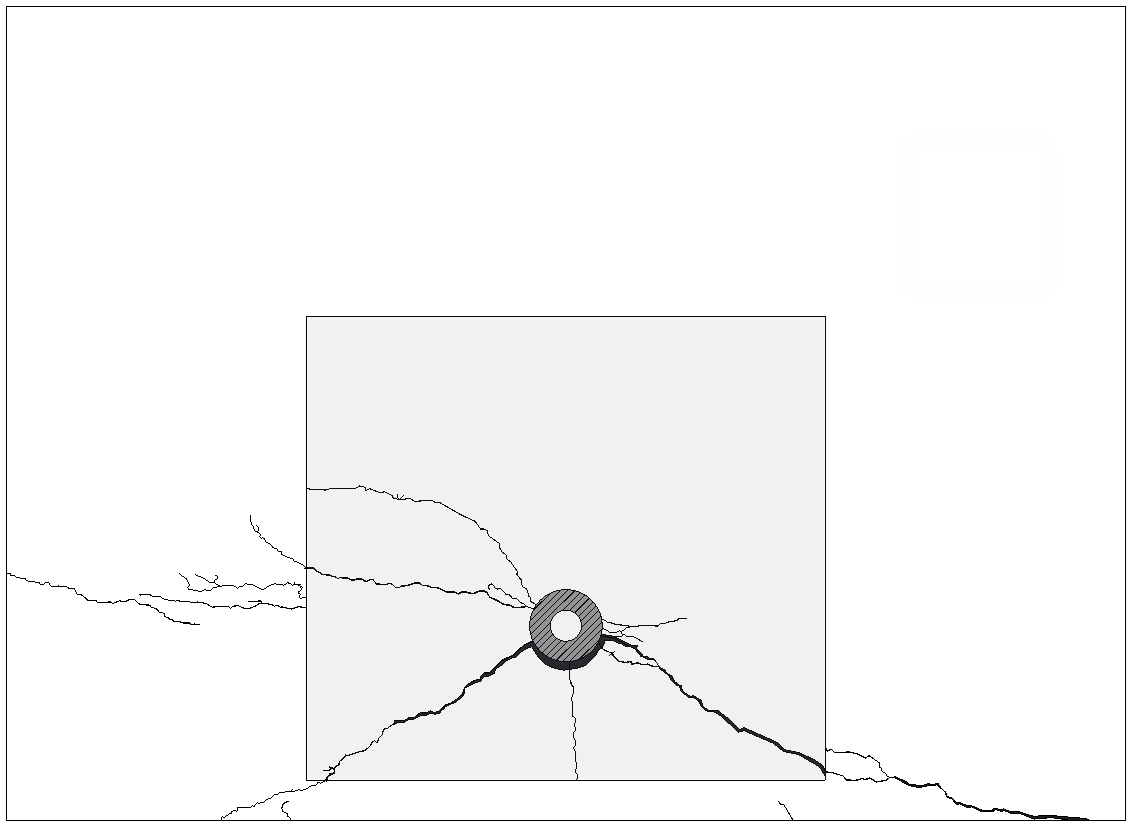

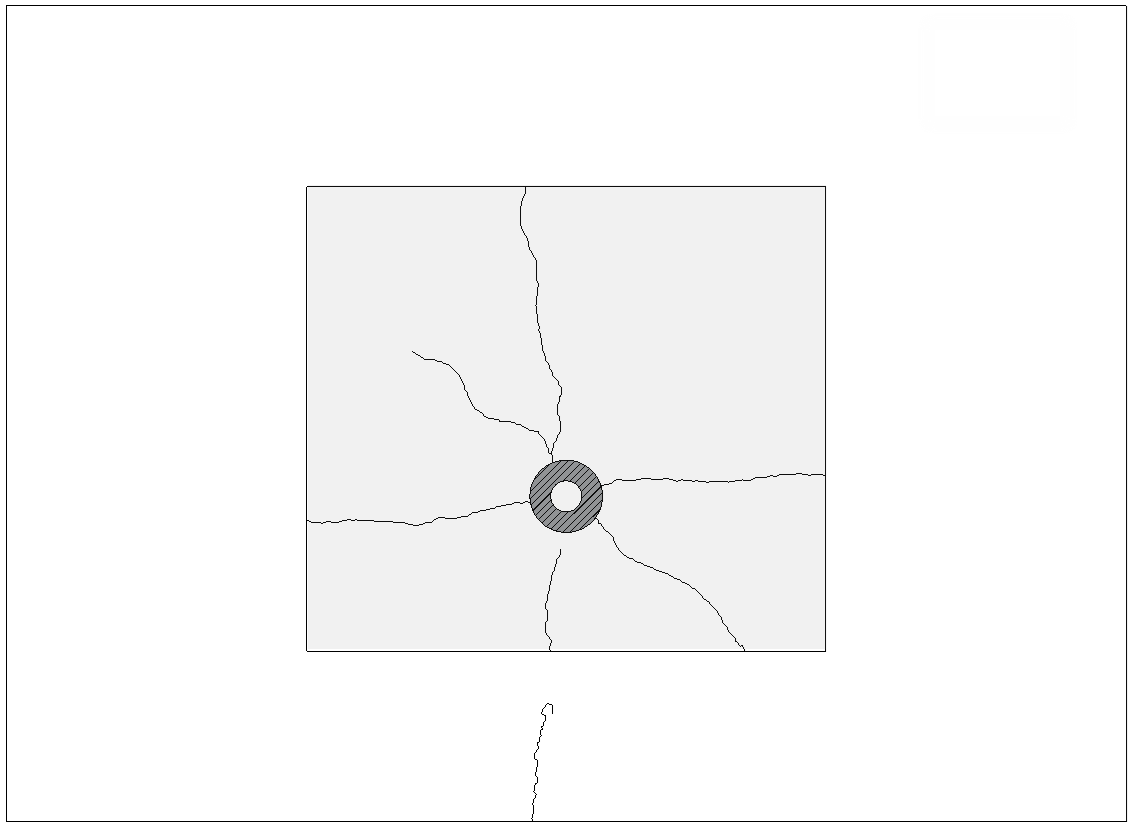


**Fig. A.13** LN-2.7d-15 **Fig. A.14** LH-4.5d-15


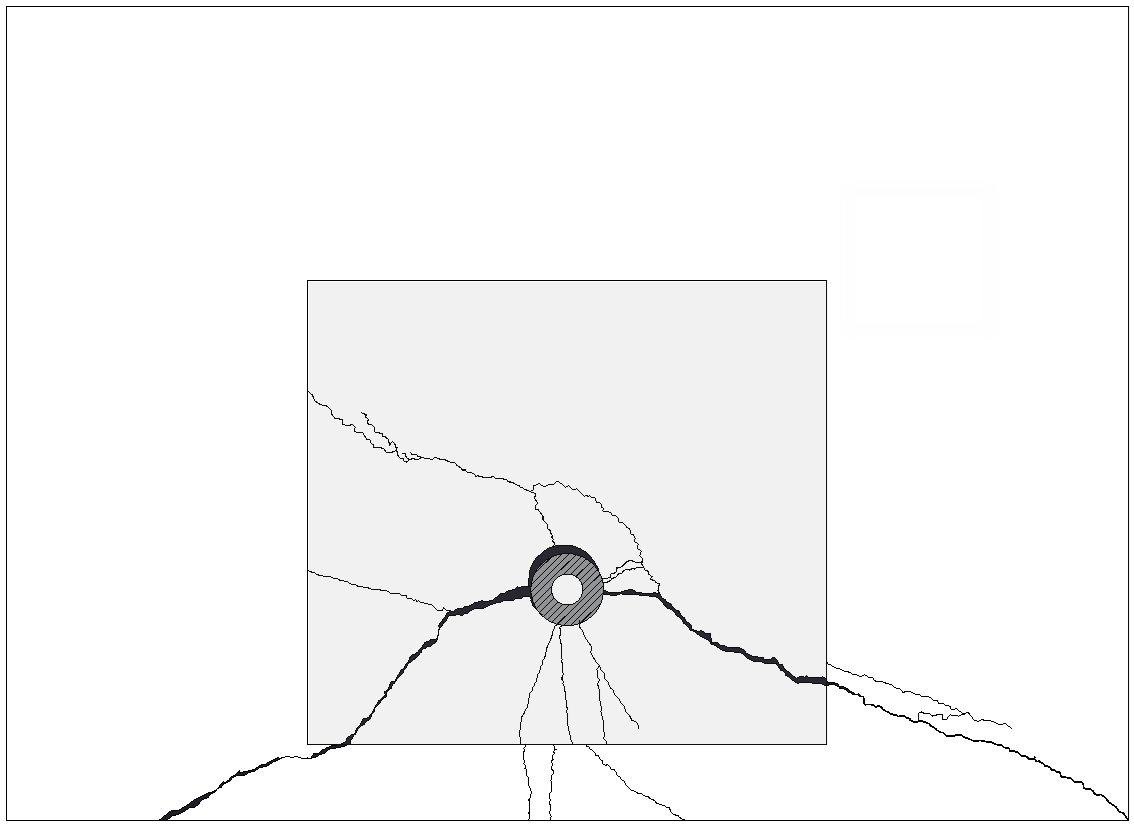

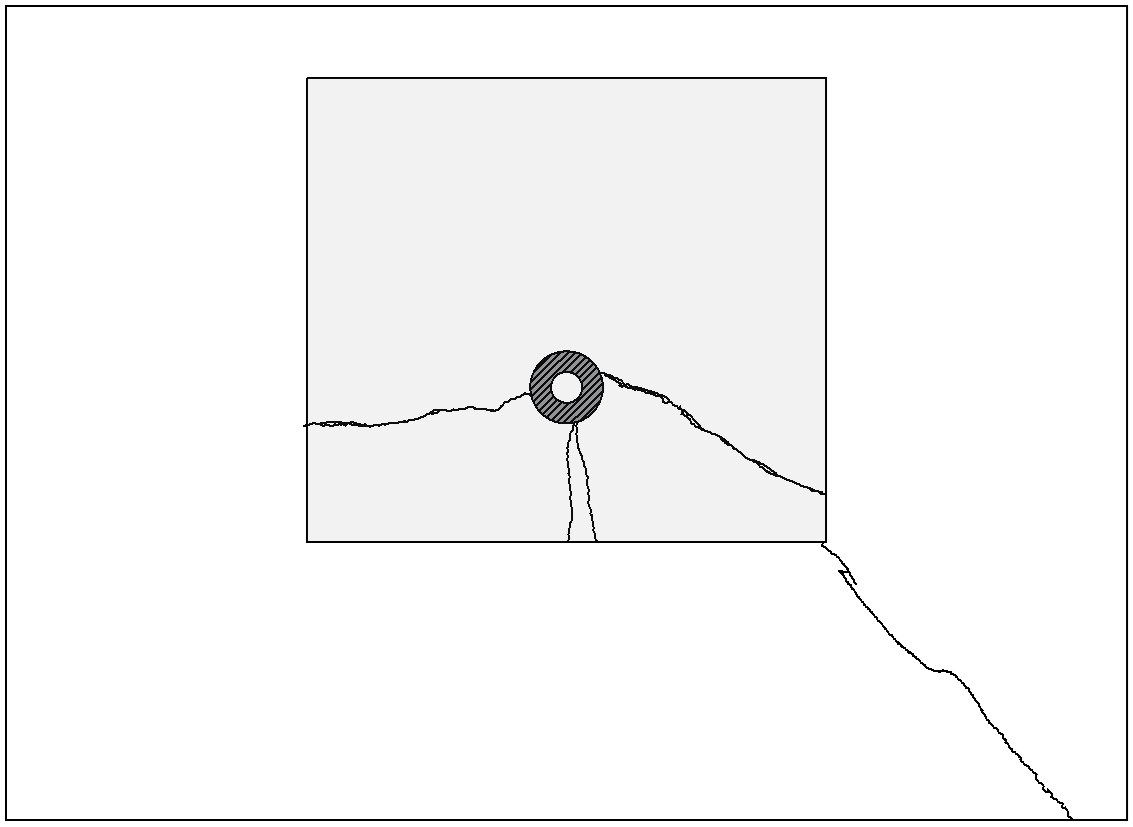


**Fig. A.15** LH-3.2d-15 **Fig. A.16** HN-6d-15


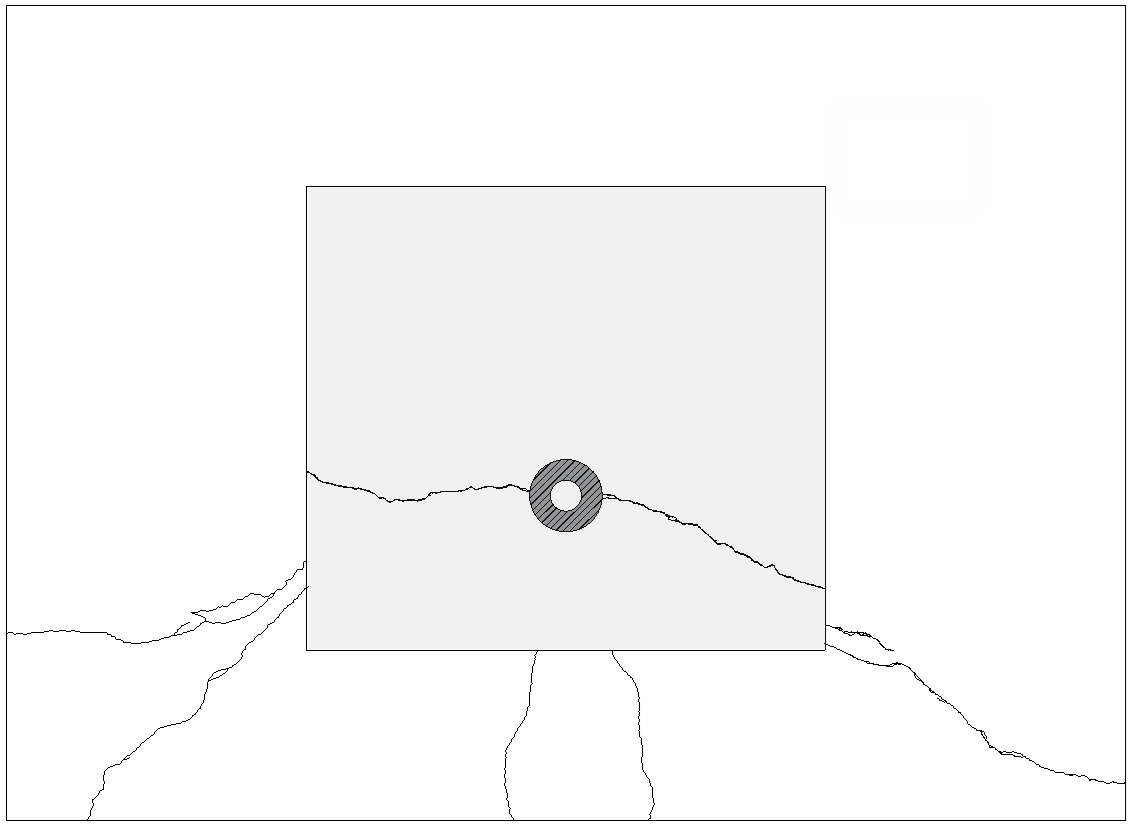

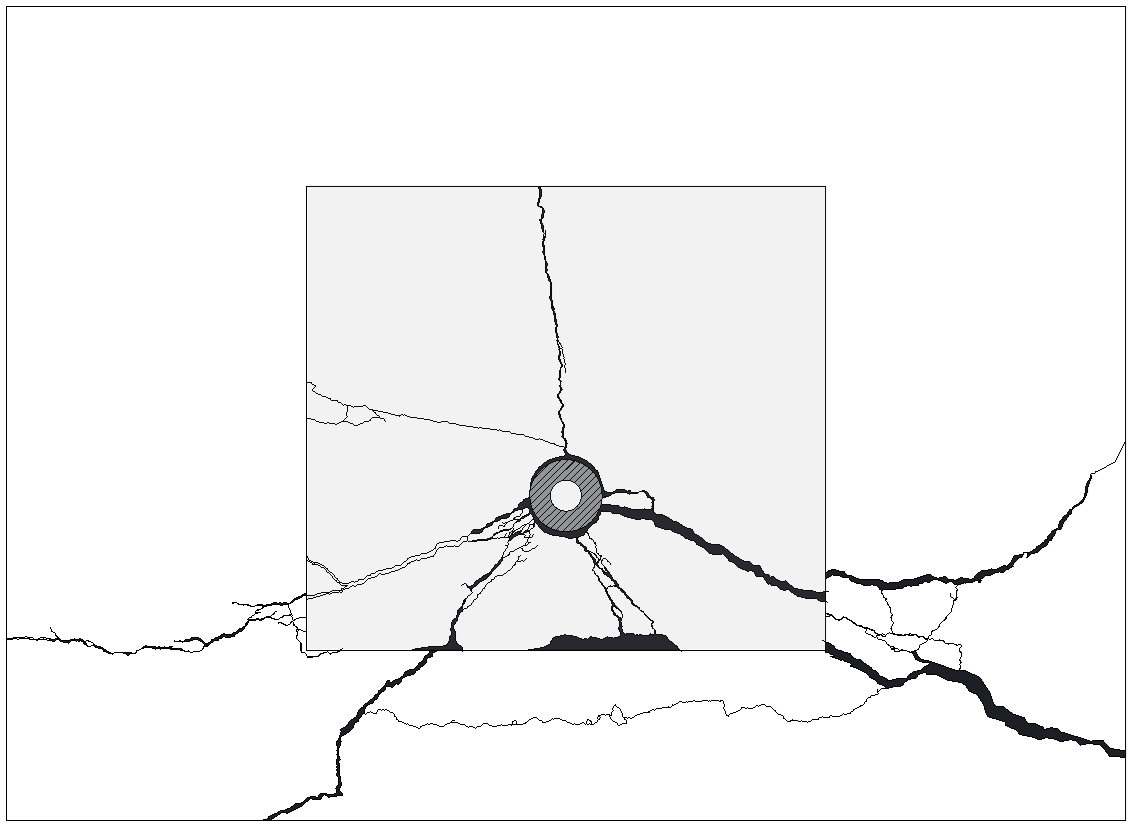


**Fig. A.17** HN-4.5d-30 **Fig. A.18** HN-4.5d-23


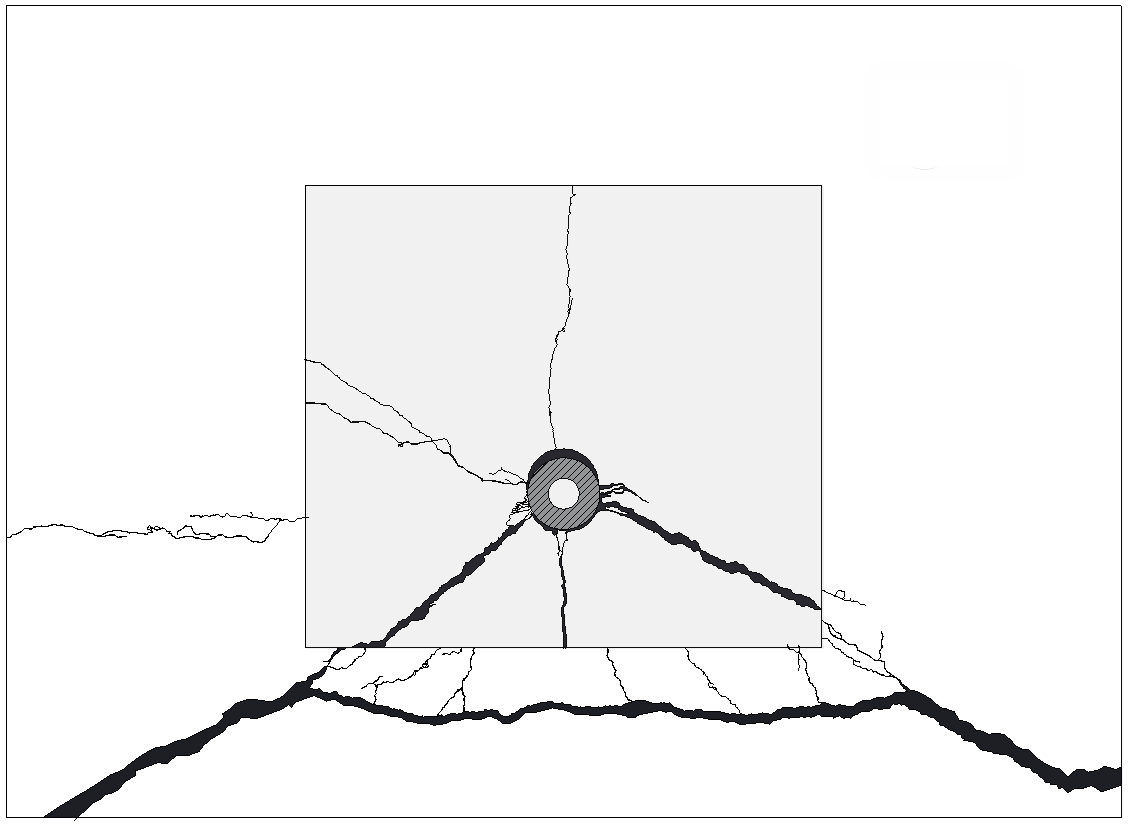

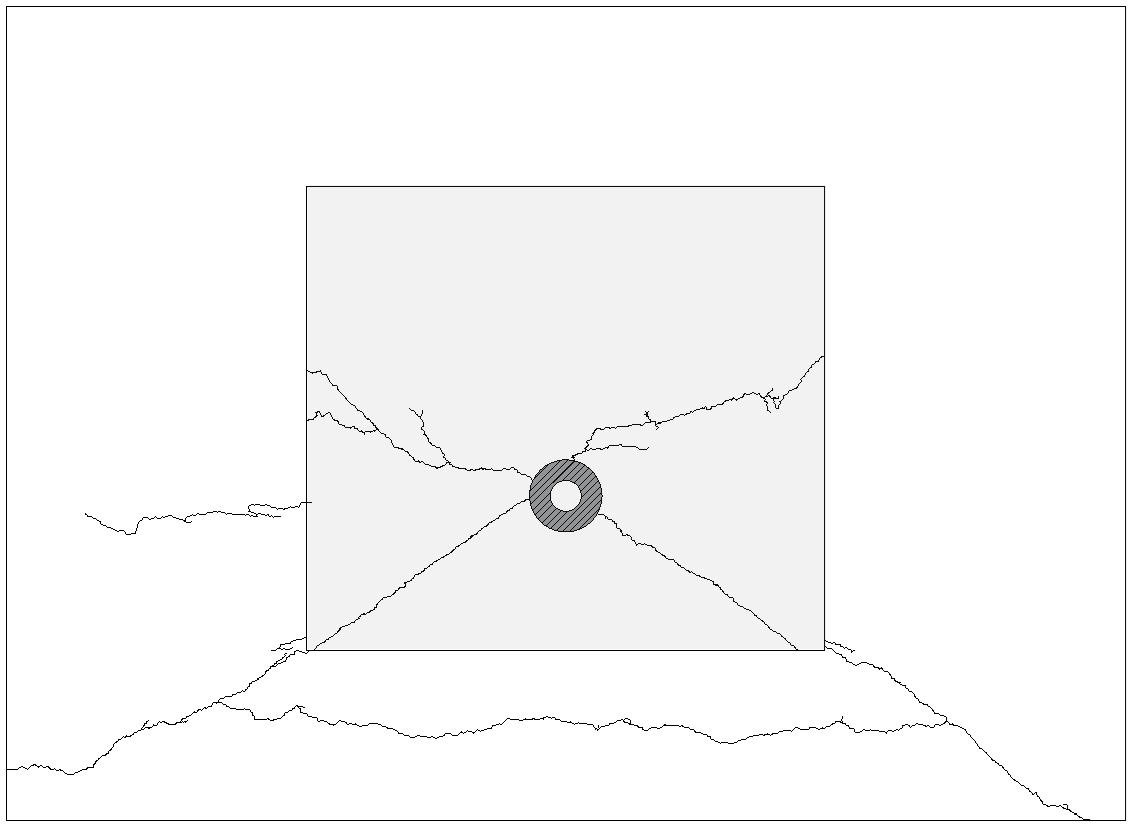


**Fig. A.19** HN-4.5d-15 **Fig. A.20** HN-4.5d-15 (2)


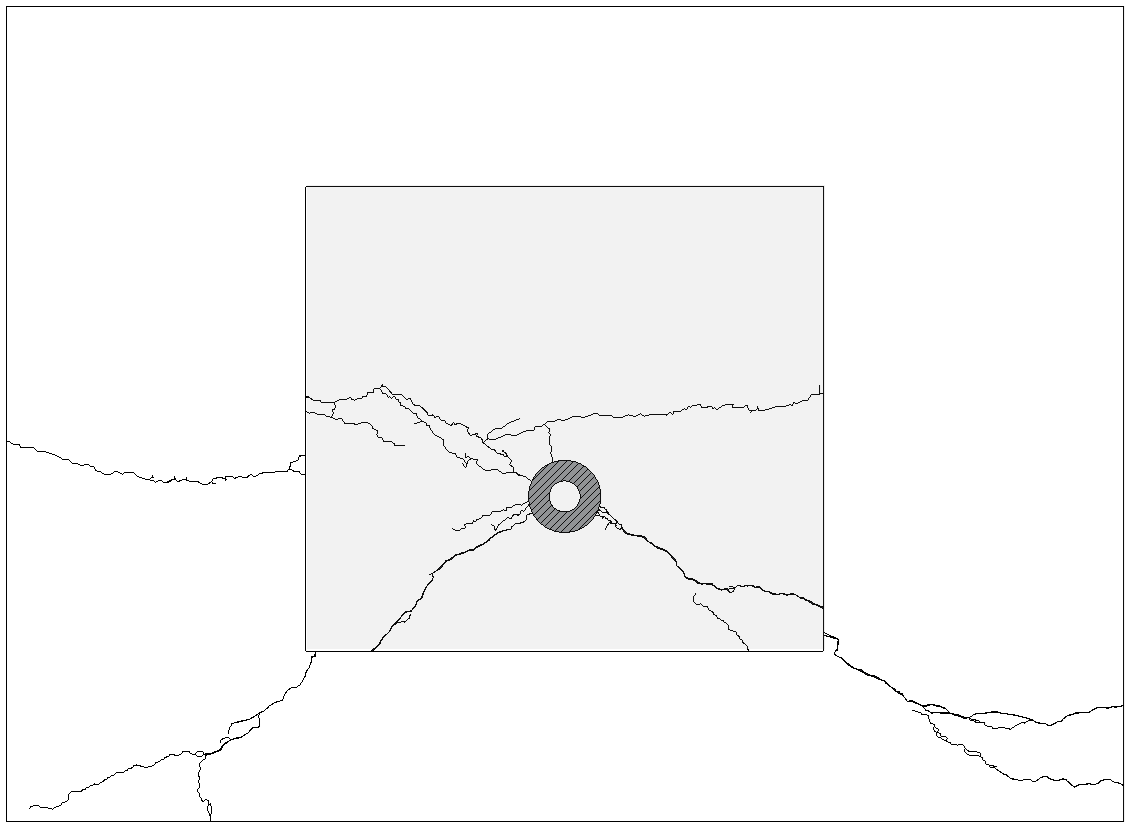


**Fig. A.21** HH-4.5d-15-

**Appendix B: Sample Calculation of LN-4.5d-15 for Models Used in Fig. 17**

***1. Contribution of concrete***

The mean concrete edge breakout resistance of the anchorage in unreinforced concrete can be calculated according to the following process [3, 12].

$$V_{Rm,c}= {V^{0}}_{Rm,c}\frac{A_{Vc}}{A_{Vco}}\psi_{ec,V}\psi_{h,V}=214.27\cdot\frac{370\cdot3\cdot315}{4.5\cdot{315}^{2}}\cdot1\cdot1.13=189.6 kN$$

where ${V^{0}}_{Rm,c}$ is the mean basic resistance in case of concrete edge failure of a single anchor, N; $\psi_{ec,V}$ is a factor to account for load eccentricity and $\psi_{h,V}$ is a factor to account for influence of member thickness;

$${V^{0}}_{Rm,c}= 1.33k_{9}d^{\alpha}{l_{e}}^{\beta}\sqrt{f_{cm}}{c_{a1}}^{1.5}=1.33\cdot2.4\cdot{70}^{0.069}\cdot{150}^{0.074}\cdot\sqrt{38.2}\cdot{315}^{1.5}=214270 N$$

$$\alpha=0.1{(l_{e}/c_{a1})}^{0.5}= 0.1{(150/315)}^{0.5}=0.069$$

$$\beta=0.1{(d/c_{a1})}^{0.2}= 0.1{(70/315)}^{0.2}=0.074$$

where $k_{9}$ is 2.4 for uncracked concrete and $f_{cm}$ is mean concrete cylinder compressive strength ($f_{ck}+8$MPa by EN 1992-1-1);

***2. Failure load calculated by EN1992-4 model***

The mean resistance $V_{Rm,re}$ of the supplementary reinforcement of anchorage failure in the concrete edge breakout body can be calculated according to following process [3, 62]. Only bars within a distance $\leq$0.75 times the edge distance from the fastener are assumed as effective reinforcement.

$$V_{Rm,re}= \sum_{n} V_{Rm,re}^{0}$$

with

$$V_{Rm,re}^{0}= \frac{l_{1}\pi d_{s}f_{bm}}{\alpha_{1}\alpha_{2}x}\leq\frac{f_{ym}A_{s,re}}{x}, N$$

where n is the number of the effective legs of the anchor reinforcement; $l_{1}$ is the anchorage length inside the theoretical breakout body, mm; $d_{s}$ is the diameter of the stirrup, mm; $f_{bm}$ is the characteristic bond strength, MPa (=$2f_{bd}); f_{bd}$ is the design bond strength according to EN1992-1-1; $f_{ym}$ is the characteristic yield strength of rebar, MPa; $A_{s,re}$ is the area of stirrup, mm^2^; $\alpha_{1}$ is 1.0 for straight rebar and $\alpha_{2}$ is the factor to consider the effect of cover on the bond strength defined as

$\alpha_{2}=1-\frac{0.15\left( c_{d}-3d_{s} \right)}{d_{s}}= 1-\frac{0.15(50-3\cdot16)}{16}$ = 1 (0.7$\leq\alpha_{2}\leq$1.0)

where $c_{d}$ is the clear cover to the reinforcement or half the clear distance to the adjacent reinforcement, mm;

$$x=\left( 1+\frac{e_{s}}{z} \right)=\left( 1+\frac{115}{0.85\cdot min(370, 2\cdot150, 2\cdot315)} \right)=1.45$$

where $e_{s}$ is the distance between reinforcement and shear load acting on plate and z is the 0.85min(depth of concrete member, 2$l_{e}$, 2$c_{a1}$)

The failure load for an anchorage with supplementary reinforcement is given as

$V_{Rm}= max(V_{Rm,c};V_{Rm,re})$ = 189.6 kN

**Table B.1** Calculation for $V_{Rm,re}$ by EN1992-4

| Location of Effective stirrup^*^  (distance from anchor, mm) | -150 | -50 | 50 | 150 |
| --- | --- | --- | --- | --- |
| $l_{1}$ (mm)^**^ | 139.85 | 201.06 | 201.06 | 139.85 |
| $d_{s}$ (mm) | 16 | 16 | 16 | 16 |
| $f_{bm}$ (MPa) | 5.99 | 5.99 | 5.99 | 5.99 |
| $\alpha_{1}$ | 1 | 1 | 1 | 1 |
| $\alpha_{2}$ | 1 | 1 | 1 | 1 |
| $x$ | 1.45 | 1.45 | 1.45 | 1.45 |
| $V_{Rm,re}^{0}$ (kN) | 0 | 41.70 | 41.70 | 0 |
| $V_{Rm,re}$ (kN) | 83.4 | | | |

* Only bars within a distance $\leq$0.75$c_{a1}$ from the fastener are assumed as effective reinforcement.

**$l_{1}$ is modified from EN1992-4 to reflect actual effective length considering both horizontal and vertical propagations of the crack at an angle of 1:1.5 because of consideration of high lever arm in the bridge bearing anchor. $l_{1}$ should be greater than 10 times the diameter of the stirrup in EN1992-4, thus effects of stirrup located at -150 mm and 150 mm were not considered.

***3. Failure load calculated by Schmid model***

The mean shear capacity of anchorage with anchor reinforcement corresponding to reinforcement failure by Schmid model can be calculated according to following process

$$V_{Rm,re}= \frac{N_{Rm,re}}{x} \geq V_{Rm,c}$$

$$N_{Rm,re}= \sum_{n} N_{Rm,re}^{0}$$

$$N_{Rm,re}^{0}= N_{Rm,hook}^{0}+ N_{Rm,bond}^{0} \leq A_{s,i}f_{ym}$$

where n is the number of the effective legs of the anchor reinforcement; $N_{Rm,hook}^{0}$ is the contribution of the hook of the stirrup; $N_{Rm,bond}^{0}$ is the contribution of the bond of one stirrup; and $A_{s,i}$ is the area of one (*i*^th^) stirrup;

$$N_{Rm,hook, i}^{0}= \psi_{1,i}\psi_{2}\psi_{3}A_{s,i}f_{ym}\left( \frac{f_{cm,cube}}{30} \right)^{0.1}, N$$

where $\psi_{1,i}$ is 0.95 for the most effective stirrups or 0.16 for other stirrups and $f_{cm,cube}$ is the mean compressive strength of concrete obtained using 150 mm cubes.

$$\psi_{2}= \left( \frac{d_{s,L}}{d_{s}} \right)^{2/3}$$

$$\psi_{3}= \left( \frac{l_{1,i}}{c_{a1}} \right)^{0.4}\cdot\left( \frac{10}{d_{s}} \right)^{0.25}$$

where $d_{s,L}$ is diameter of outermost reinforcement perpendicular to anchor reinforcement and $l_{1,i}$ is the bond length of the *i*^th^ stirrup.

$$N_{Rm,bond, i}^{0}= \pi d_{s}l_{1,i}^{'}f_{bm}/\alpha_{2}, N$$

where $l_{1,i}^{'}= l_{1,i}-4d_{s}$.

**Table B.2** Calculation for $V_{Rm,re}$ by Schmid model

| Location of Effective stirrup^*^  (distance from anchor, mm) | -250 | -150 | -50 | 50 | 150 | 250 |
| --- | --- | --- | --- | --- | --- | --- |
| $\psi_{1,i}$ | 0.16 | 0.16 | 0.95 | 0.95 | 0.16 | 0.16 |
| $\psi_{2}$ | 1 | 1 | 1 | 1 | 1 | 1 |
| $\psi_{3}$ | 0.51 | 0.64 | 0.74 | 0.74 | 0.64 | 0.51 |
| $l_{1,i}$ (mm) | 78.63 | 139.85 | 201.06 | 201.06 | 139.85 | 78.63 |
| $f_{ym}$ (MPa) | 446.13 | 446.13 | 446.13 | 446.13 | 446.13 | 446.13 |
| $f_{ck}$ (MPa) | 30.20 | 30.20 | 30.20 | 30.20 | 30.20 | 30.20 |
| $f_{cm,cube}$ (MPa) | 47.75 | 47.75 | 47.75 | 47.75 | 47.75 | 47.75 |
| $N_{Rm,hook, i}^{0}$ (kN) | 7.58 | 9.54 | 65.51 | 65.51 | 9.54 | 7.58 |
| $l_{1,i}^{'}$ (mm) | 14.63 | 75.85 | 137.06 | 137.06 | 75.85 | 14.63 |
| $f_{bm}$ (MPa) | 5.99 | 5.99 | 5.99 | 5.99 | 5.99 | 5.99 |
| $\alpha_{2}$ | 1 | 1 | 1 | 1 | 1 | 1 |
| $N_{Rm,bond,i}^{0}$ (kN) | 4.40 | 22.82 | 41.25 | 41.25 | 22.82 | 4.40 |
| $N_{Rm,re}^{0}$ (kN) | 11.98 | 32.37 | 88.60^**^ | 88.60^**^ | 32.37 | 11.98 |
| $N_{Rm,re}$ (kN) | 265.9 | | | | | |
| $V_{Rm,re}$ (kN) | 183.3 | | | | | |

* Only bars with $l_{1,i}\geq4d_{s}$ in the theoretical breakout body are assumed as effective reinforcement.

** The value calculated by $A_{s,i}f_{ym}$ is used

The failure load of anchorage with anchor reinforcement corresponding to reinforcement failure by Schmid is 189.6 kN.

***4. Failure load calculated by Sharma model***

The failure load of anchorage with anchor reinforcement corresponding to reinforcement failure by Sharma model can be calculated as the failure load corresponding to concrete edge failure in unreinforced concrete plus the load corresponding to failure of reinforcement when uplift of baseplate is not restrained, as follows:

$$V_{Rm}= 0.5V_{Rm,c}+ V_{Rm,re}\leq V_{Rm,st}$$

$$V_{Rm,st}= V_{Rm,c}\psi_{st,V}= V_{Rm,c}\cdot(2.75-1.17X/c_{a1})$$

where $V_{Rm,st}$ is the failure load in case of strut failure and X is the distance between anchor and stirrup.

For LN-4.5d-15, all of the stirrups did not yield, thus the effectiveness factor $\psi_{1}$ is the same as the factor of the Schmid model. Therefore, $V_{Rm}$ can be calculated as follows:

$$V_{Rm}= 0.5V_{Rm,c}+ V_{Rm,re}=0.5\cdot189.6+183.3=278.1 kN\leq486.2 kN$$
